# Effect of Dexamethasone on Nocturnal Oxygenation in Lowlanders With Chronic Obstructive Pulmonary Disease Traveling to 3100 Meters: A Randomized Clinical Trial

**DOI:** 10.1001/jamanetworkopen.2019.0067

**Published:** 2019-02-22

**Authors:** Michael Furian, Mona Lichtblau, Sayaka S. Aeschbacher, Bermet Estebesova, Berik Emilov, Ulan Sheraliev, Nuriddin H. Marazhapov, Maamed Mademilov, Batyr Osmonov, Maya Bisang, Stefanie Ulrich, Tsogyal D. Latshang, Silvia Ulrich, Talant M. Sooronbaev, Konrad E. Bloch

**Affiliations:** 1 Department of Respiratory Medicine, University Hospital Zurich, Zurich, Switzerland; 2 Department of Respiratory Medicine, National Center for Cardiology and Internal Medicine, Bishkek, Kyrgyz Republic

## Abstract

**Importance:**

During mountain travel, patients with chronic obstructive pulmonary disease (COPD) are at risk of experiencing severe hypoxemia, in particular, during sleep.

**Objective:**

To evaluate whether preventive dexamethasone treatment improves nocturnal oxygenation in lowlanders with COPD at 3100 m.

**Design, Setting, and Participants:**

A randomized, placebo-controlled, double-blind, parallel trial was performed from May 1 to August 31, 2015, in 118 patients with COPD (forced expiratory volume in the first second of expiration [FEV_1_] >50% predicted, pulse oximetry at 760 m ≥92%) who were living at altitudes below 800 m. The study was conducted at a university hospital (760 m) and high-altitude clinic (3100 m) in Tuja-Ashu, Kyrgyz Republic. Patients underwent baseline evaluation at 760 m, were taken by bus to the clinic at 3100 m, and remained at the clinic for 2 days and nights. Participants were randomized 1:1 to receive either dexamethasone, 4 mg, orally twice daily or placebo starting 24 hours before ascent and while staying at 3100 m. Data analysis was performed from September 1, 2015, to December 31, 2016.

**Interventions:**

Dexamethasone, 4 mg, orally twice daily (dexamethasone total daily dose, 8 mg) or placebo starting 24 hours before ascent and while staying at 3100 m.

**Main Outcomes and Measures:**

Difference in altitude-induced change in nocturnal mean oxygen saturation measured by pulse oximetry (Spo_2_) during night 1 at 3100 m between patients receiving dexamethasone and those receiving placebo was the primary outcome and was analyzed according to the intention-to-treat principle. Other outcomes were apnea/hypopnea index (AHI) (mean number of apneas/hypopneas per hour of time in bed), subjective sleep quality measured by a visual analog scale (range, 0 [extremely bad] to 100 [excellent]), and clinical evaluations.

**Results:**

Among the 118 patients included, 18 (15.3%) were women; the median (interquartile range [IQR]) age was 58 (52-63) years; and FEV_1_ was 91% predicted (IQR, 73%-103%). In 58 patients receiving placebo, median nocturnal Spo_2_ at 760 m was 92% (IQR, 91%-93%) and AHI was 20.5 events/h (IQR, 12.3-48.1); during night 1 at 3100 m, Spo_2_ was 84% (IQR, 83%-85%) and AHI was 39.4 events/h (IQR, 19.3-66.2) (*P* < .001 both comparisons vs 760 m). In 60 patients receiving dexamethasone, Spo_2_ at 760 m was 92% (IQR, 91%-93%) and AHI was 25.9 events/h (IQR, 16.3-37.1); during night 1 at 3100 m, Spo_2_ was 86% (IQR, 84%-88%) (*P* < .001 vs 760 m) and AHI was 24.7 events/h (IQR, 13.2-33.7) (*P* = .99 vs 760 m). Altitude-induced decreases in Spo_2_ during night 1 were mitigated by dexamethasone vs placebo by a mean of 3% (95% CI, 2%-3%), and increases in AHI were reduced by 18.7 events/h (95% CI, 12.0-25.3). Similar effects were observed during night 2. Subjective sleep quality was improved with dexamethasone during night 2 by 12% (95% CI, 0%-23%). Sixteen (27.6%) patients using dexamethasone had asymptomatic hyperglycemia.

**Conclusions and Relevance:**

In lowlanders in Central Asia with COPD traveling to a high altitude, preventive dexamethasone treatment improved nocturnal oxygen saturation, sleep apnea, and subjective sleep quality.

**Trial Registration:**

ClinicalTrials.gov Identifier: NCT02450994

## Key Points

**Question:**

Does preventive dexamethasone treatment mitigate altitude-related nocturnal hypoxemia in lowlanders with chronic obstructive pulmonary disease traveling to a high altitude?

**Findings:**

In this randomized clinical trial of 118 patients with chronic obstructive pulmonary disease who lived below 800 m, dexamethasone, 4 mg, orally twice daily, starting 24 hours before ascent and while staying in a clinic at 3100 m, significantly mitigated the altitude-induced decrease in mean arterial oxygen saturation during 2 nights at 3100 m by 2% to 3%. In addition, dexamethasone therapy prevented emergence of central sleep apnea and improved subjective sleep quality.

**Meaning:**

Patients with chronic obstructive pulmonary disease traveling to a high altitude may benefit from preventive dexamethasone treatment in terms of nocturnal oxygenation and sleep quality.

## Teaser

This randomized clinical trial examines the effect of dexamethasone therapy on nocturnal oxygenation in lowlanders with chronic obstructive pulmonary disease (COPD) who travel to a high altitude.

## Introduction

Worldwide, millions of persons live in or travel to mountain areas.^1^ Even though moderate hypobaric hypoxia at altitudes of 1500 to 3500 m is generally well tolerated by healthy individuals, nocturnal hypoxemia, periodic breathing, disturbances of sleep structure, and impairment of subjective sleep quality are commonly noticed.^2^ Patients with preexisting respiratory conditions, such as obstructive sleep apnea syndrome^3^ or chronic obstructive pulmonary disease (COPD),^4^ seem to be particularly susceptible to altitude-related hypoxemia, sleep, and breathing disturbances.^5^ In patients with obstructive sleep apnea syndrome, continuous positive airway pressure therapy combined with acetazolamide was effective in preventing exacerbation of sleep apnea and improving hypoxemia during altitude sojourns.^6^ In patients with COPD, mechanical ventilatory constraints combined with the stimulation of ventilation by acetazolamide and hypobaric hypoxia at high altitudes may promote dyspnea, but to our knowledge, this has not been investigated.^7^

The use of supplemental oxygen is hampered for logistical reasons and because its use is cumbersome and difficult for travelers with COPD. Therefore, other means to prevent altitude-related adverse health effects in patients with COPD are warranted. In healthy mountaineers, dexamethasone, a drug with potent glucocorticoid action, has been shown to prevent acute mountain sickness (AMS),^8,9^ reduce pulmonary artery pressure, and stimulate ventilation in individuals susceptible to high-altitude pulmonary edema.^10^ In patients with COPD, glucocorticoids are used to treat exacerbations.^11^ The present randomized, placebo-controlled trial was performed in lowlanders in Central Asia with mild to moderate COPD to evaluate the hypothesis that preventive dexamethasone treatment would mitigate nocturnal hypoxemia, periodic breathing, and impairments of sleep quality during a stay at high altitude.

## Methods

### Design and Setting

This study was part of a project investigating prevention of altitude-related adverse health effects in patients with COPD. Patient characteristics, data on AMS, and disturbances of postural control have been reported.^12,13^ The present analysis of secondary end points of the randomized, double-blind, placebo-controlled, parallel-group trial evaluates the efficacy of dexamethasone in preventing altitude-related nocturnal hypoxemia in lowlanders with COPD traveling to and staying for 2 nights at 3100 m. The protocol was approved by the ethics committee of the National Center for Cardiology and Internal Medicine and endorsed by the Cantonal Ethics Committee Zurich, Switzerland, and is available in Supplement 1. Participants gave written informed consent; there was no financial compensation. This study followed the Consolidated Standards of Reporting Trials (CONSORT) reporting guideline.^14^

From May 1 to August 31, 2015, participants underwent baseline examinations at the National Center for Cardiology and Internal Medicine, Bishkek, Kyrgyz Republic, at 760 m (mean barometric pressure, 700 mm Hg), then traveled by minibus within 3 to 5 hours to the High-Altitude Clinic, Tuja-Ashu, Kyrgyz Republic, at 3100 m (barometric pressure, 545 mm Hg) and stayed there for 2 nights. On the day before and during altitude sojourns, participants received dexamethasone or placebo according to randomization. For safety reasons, participants with relevant intercurrent illness, severe hypoxemia (pulse oximetry [Spo_2_] <75% for >30 minutes or <70% for >15 minutes), or requesting to descend to lower altitude because of discomfort received supplemental oxygen, were withdrawn from the study, and were relocated to lower altitude.

### Participants

Among outpatients of the National Center for Cardiology and Internal Medicine and other clinics of the Bishkek area, men and women aged 20 to 75 years, with a diagnosis of grade 1 or 2 COPD according to guidelines of the Global Initiative for Chronic Obstructive Lung Disease (GOLD) (grade 1, mild: postbronchodilator forced expiratory volume in the first second of expiration [FEV_1_]/forced vital capacity [FVC] <0.7, FEV_1 _≥80% predicted; grade 2, moderate: FEV_1_/FVC <0.7, FEV_1_ 50%-79% predicted)^11^ and living at an altitude lower than 800 m were invited to participate. Exclusion criteria were more than mild hypoxemia at 760 m (Spo_2 _<92%); exacerbation of COPD within 3 months before the study; diabetes; any uncontrolled cardiovascular, neurologic, or psychiatric disease; heavy smoking (>20 cigarettes per day); and a stay at an altitude higher than 1000 m in the past month.

### Interventions

Dexamethasone, 4 mg, capsules or identical-looking placebo capsules were administered at breakfast and dinner (dexamethasone total daily dose, 8 mg) under supervision of one of the investigators starting 24 hours before ascent and during the stay at 3100 m.

### Assessments

A medical history was obtained and a clinical examination was performed. Symptoms were evaluated by the COPD Assessment Test. The test contains 8 categories about COPD symptoms; each category ranges from 0 (I am very happy) to 5 (I am very sad). A score of 5 points represents the upper limit of normal; greater than 5 and lower than 10 indicates low effect and most days are good; greater than 10 and less than 20 indicates medium effect and COPD is one of the most important problems that they have; and greater than 20 indicates high effect and COPD stops them from doing most things that they want to do.^15^ Respiratory sleep studies (AlicePDx; Philips AG Respironics), including Spo_2_, nasal cannula pressure swings, thoracic and abdominal movements, snoring, electrocardiogram, and body position, were performed. Indices of oxygenation and the mean number of apneas/hypopneas per hour of time in bed (apnea/hypopnea index [AHI]) were determined. Cerebral tissue oxygenation was monitored with near-infrared spectroscopy sensors (NIRO 200NX; Hamamatsu Photonics) placed bilaterally, high on the forehead.^16^ Subjective sleep quality was rated on a 100-mm visual analog scale ranging from 0 (extremely bad) to 100 (excellent), and insomnia was evaluated by asking participants to estimate the number of awakenings and time spent awake at night.^17^ The Karolinska Sleepiness Scale was administered (score range from 1 [extremely alert] to 9 [very sleepy, great effort to keep alert, fighting sleep]).^18^ Vital signs, spirometry,^19^ and arterial blood gas analysis were obtained. The reaction time was assessed by the psychomotor vigilance test.^20^ Assessments are further explained in eMethods in Supplement 2.

### Outcomes

The primary outcome was the between-group difference in altitude-induced changes in mean nocturnal Spo_2_ during the first night at 3100 m. Secondary outcomes were effects of altitude and dexamethasone on various clinical and physiologic measures.

### Sample Size

Sample size estimation based on the trial evaluating effects of dexamethasone on AMS indicated a minimal number of 100 participants.^12^ The present trial was powered with 80% to detect a mean (SD) minimal difference in Spo_2_ (primary outcome) of 2% (3.5%)^5^ and, in AHI, an important secondary outcome, of 10 (17) events/h, with a 2-sided significance level of α = .05.

### Randomization

Patients were randomized by an independent person 1:1 to dexamethasone or placebo in blocks of 5, minimizing for sex, age 50 years or younger and older than 50 years, and FEV_1_ less than 80% predicted or 80% predicted and higher, using a computer algorithm.

### Blinding

Identical-looking dexamethasone and placebo capsules were dispensed and labeled with a concealed code by an independent pharmacist. Participants and investigators were blinded to the study drug until completion of data analysis.

### Statistical Analysis

Data analysis was performed from September 1, 2015, to December 31, 2016. Data are presented as medians (interquartile ranges) and mean differences (95% CIs). The primary outcome was analyzed according to the intention-to-treat principle. Missing values were filled by 20 imputations using regression models with chained equations^21^ including the following predictors at 760 m: drug assignment, study night, anthropometrics, daytime Spo_2_, FEV_1_% predicted, and body mass index. Secondary outcomes were analyzed by the per-protocol approach without replacing missing values. Between-group comparisons were performed by Mann-Whitney tests and, by computing mean differences with 95% CIs, intragroup comparisons were performed by Wilcoxon signed rank tests. Regression analyses were performed to elucidate independent predictors of outcomes at 3100 m. A 2-tailed *P* value <.05 was considered statistically significant. The statistical analysis plan is available in Supplement 1.

## Results

**Figure 1.**
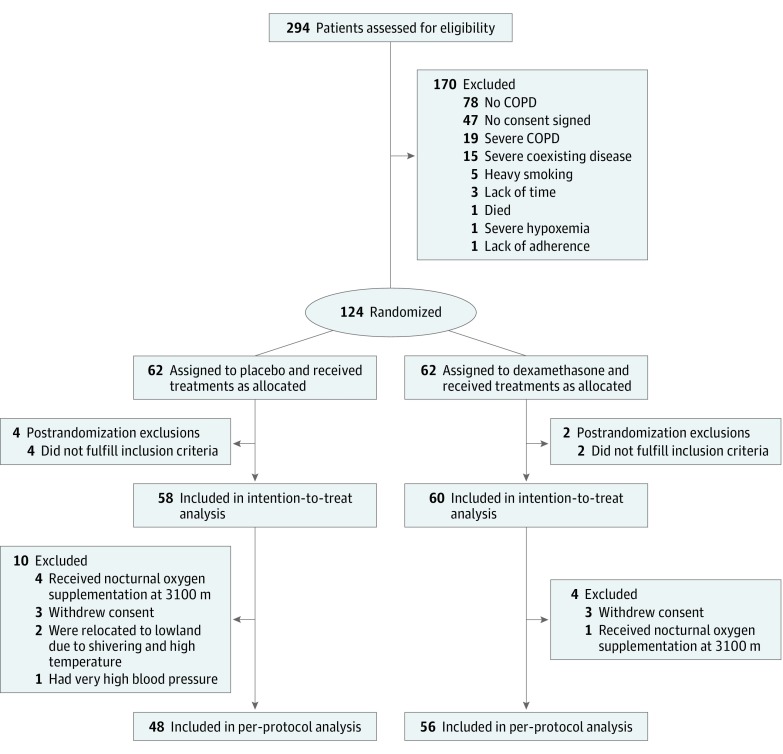
CONSORT Flowchart COPD indicates chronic obstructive pulmonary disease.

A total of 294 individuals were screened, and 124 were randomized (Figure 1); 6 patients were excluded post randomization; 4 had severe coexisting disease and 2 were living above 1500 m. The intention-to-treat analysis included 58 patients randomized to placebo and 60 randomized to dexamethasone. Of these, 10 of 58 (17.2%) patients in the placebo group and 4 of 60 (6.7%) patients in the dexamethasone group (*P* = .09 between dexamethasone and placebo, Fisher exact test) had incomplete data for various reasons (Figure 1). Seven of 58 (12.1%) patients assigned to placebo and 1 of 60 (1.7%) assigned to dexamethasone (*P* = .03 between dexamethasone and placebo, Fisher exact test) could not stay both nights at 3100 m because they required oxygen and relocation to a lower altitude according to safety rules. Adverse health effects, such as excessive hypoxemia, elevated blood pressure, or discomfort for other reasons, resolved within a few hours without sequelae. Among the 118 patients included, 18 (15.3%) were women; the median (interquartile range [IQR]) age was 58 (52-63) years; and FEV_1_ was 91% predicted (IQR, 73%-103%). Further characteristics of patients receiving dexamethasone and placebo were similar (Table 1).

**Table 1.** Characteristics of the Intention-to-Treat Population Assessed at 760 m

Variable	Group		
	All (N = 118)	Placebo (n = 58)	Dexamethasone (n = 60)
Men, No. (%)	100 (84.7)	50 (86.2)	50 (83.3)
Women, No. (%)	18 (15.3)	8 (13.8)	10 (16.7)
Age, median (IQR), y	58 (52-63)	60 (53-64)	57 (50-62)
BMI, median (IQR)	25.6 (22.8-27.7)	25.5 (22.7-27.5)	25.7 (23.4-27.7)
FEV_1_, median (IQR), L	2.6 (2.1-3.0)	2.7 (2.2-3.0)	2.5 (1.9-3.0)
% Predicted	91 (73-103)	94 (76-103)	86 (70-104)
FVC, median (IQR), L	4.2 (3.6-4.7)	4.2 (3.6-4.9)	4.1 (3.4-4.5)
% Predicted	117 (101-129)	118 (104-134)	117 (99-125)
FEV_1_/FVC, median (IQR)	0.64 (0.57-0.68)	0.65 (0.60-0.68)	0.63 (0.56-0.66)
COPD, GOLD grade, No. (%)^a^			
1	78 (66.1)	41 (70.7)	37 (61.7)
2	40 (33.9)	17 (29.3)	23 (38.3)
Pulse oximetry, median (IQR), %	95 (94-96)	95 (95-96)	95 (94-96)
Smoking, median (IQR), pack-years	20 (0-34)	24 (2-35)	20 (0-34)
NYHA classification, median (IQR)^b^	2 (1-2)	2 (1-2)	2 (1-2)
COPD Assessment Test score, median (IQR)^c^	7 (5-11)	7 (3-11)	7 (5-11)
Regular medication, No. (%)			
Inhaled			
β-Adrenergics	2 (1.7)	1 (1.7)	1 (1.7)
Anticholinergics	5 (4.2)	3 (5.2)	2 (3.3)
Corticosteroids	1 (0.8)	1 (1.7)	0
Antihypertensives	13 (11.0)	8 (13.8)	5 (8.3)
β-Blockers	5 (4.2)	2 (3.4)	3 (5.0)
Antidiabetics	1 (0.8)	1 (1.7)	0

Abbreviations: BMI, body mass index (calculated as weight in kilograms divided by height in meters squared); COPD, chronic obstructive pulmonary disease; FEV_1_, forced expiratory volume in the first second of expiration; FVC, forced vital capacity; GOLD, Global Initiative for Obstructive Lung Disease; IQR, interquartile range; NYHA, New York Heart Association.

^a^ GOLD grade 1, mild: postbronchodilator FEV_1_/FVC less than 0.7, FEV_1_ 80% or higher than predicted; grade 2, moderate: FEV_1_/FVC less than 0.7, FEV_1_ 50% to 79% predicted.

^b^ New York Heart Association classification ranging from 1 (no symptoms and no limitations) to 4 (severe limitations).

^c^ The COPD Assessment Test contains 8 categories about COPD symptoms; each category ranges from 0 (I am very happy) to 5 (I am very sad). A score of 5 points represents the upper limit of normal; greater than 5 and lower than 10 indicates low effect and most days are good; greater than 10 and less than 20 indicates medium effect and COPD is one of the most important problems that they have; and greater than 20 indicates high effect and COPD stops them from doing most things that they want to do.

**Figure 2.**
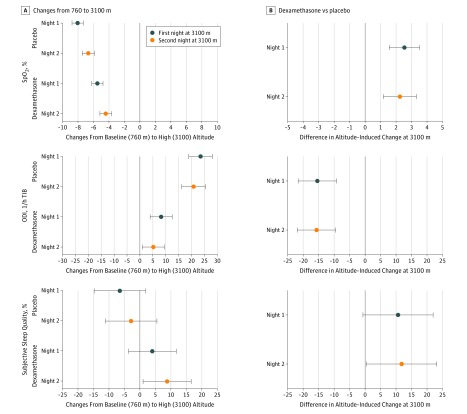
Effect of Altitude and Dexamethasone on Clinical and Physiologic Outcomes A, Mean differences in altitude-induced changes in the first and second nights at 3100 m compared with the corresponding baseline examination at 760 m in patients receiving dexamethasone and placebo. For the top graph, negative changes favor 760 m. For the middle graph, positive changes favor 760 m. For the bottom graph, negative changes favor 760 m. B, Mean differences in altitude-induced changes measured at 3100 m between patients receiving dexamethasone and placebo (treatment effect of dexamethasone). For the top graph, positive changes favor dexamethasone. For the middle graph, negative changes favor dexamethasone. For the bottom graph, positive changes favor dexamethasone. Subjective sleep quality was assessed by a visual analog scale (range, 0 [extremely bad] to 100 [excellent]). Error bars indicate 95% CI. ODI indicates oxygen desaturation index >3% dips in arterial oxygen saturation; Spo_2_, mean nocturnal oxygen saturation assessed by pulse oximetry; and TIB, time in bed.

Table 2 and Figure 2 summarize the effects of altitude travel and dexamethasone on outcomes assessed during sleep studies. eTable 1 and the eFigure in Supplement 2 provide additional details. The mean nocturnal Spo_2_ was significantly reduced during nights 1 (84%; IQR, 83%-85%) and 2 (86%; IQR, 84%-87%) at 3100 m compared with 760 m (92%; IQR, 91%-93%). Mean reduction with altitude in nights 1 and 2 was 8% (95% CI, 7%-9%) and 7% (95% CI, 6%-7%) in patients who received placebo. Corresponding mean nocturnal Spo_2_ values in patients receiving dexamethasone were 92% (IQR, 91%-93%) at 760 m, and 86% (IQR, 84%-88%) on night 1 and 87% (IQR, 86%-89%) on night 2 at 3100 m. Therefore, mean reductions with altitude during nights 1 and 2 were 5% (95% CI, 5%-6%) and 4% (95% CI, 4%-5%). Thus, dexamethasone attenuated the altitude-induced drop in Spo_2_ by a mean of 3% (95% CI, 2%-3%) during the first night and by 2% (95% CI, 1%-3%) during the second night at 3100 m (Figure 2, Table 2). In night 1 at 3100 m, patients receiving dexamethasone spent 15% (95% CI, 6%-23%) less time with Spo_2_ lower than 90% compared with patients receiving placebo and in night 2 at 3100 m they spent 22% (95% CI, 13%-30%) less time with Spo_2_ lower than 90% compared with patients receiving placebo (Table 2). Thirty-two patients (55.2%) receiving placebo and 15 of those receiving dexamethasone (25.0%) (χ^2^ test, *P* = .001) had a mean nocturnal Spo_2_ of less than 85%, which is a degree of hypoxemia considered to indicate the need for in-flight oxygen if reached during a hypoxic challenge test performed in fitness-to-fly assessment.^22^

**Table 2.** Sleep Studies and Subjective Sleep Assessment

Variable	Median (IQR)						Treatment Effect at 3100 m, Mean Difference (95% CI)	
	Placebo Group (n = 48)			Dexamethasone Group (n = 56)				
	760 m	3100 m		760 m	3100 m		Night 1	Night 2
		Night 1	Night 2		Night 1	Night 2		
Time in bed, min	545 (527 to 557)	513 (496 to 529)^a^	507 (484 to 521)^a^	533 (502 to 561)	517 (506 to 528)^a^	518 (502 to 530)^a^^,^^b^	15 (1 to 29)	20 (6 to 34)
Mean nocturnal Spo_2_, %	92 (91 to 93)	84 (83 to 85)^a^	86 (84-87)^a^	92 (91 to 93)	86 (84 to 88)^a^^,^^b^	87 (86 to 89)^a^^,^^b^	3 (2 to 3)	2 (1 to 3)
Time with Spo_2 _<90%, % of time in bed	2 (0 to 13)	98 (97 to 99)^a^	96 (91 to 99)^a^	5 (1 to 26)	96 (90 to 98)^a^^,^^b^	92 (59 to 97)^a^^,^^b^	−15 (−23 to −6)	−22 (−30 to −13)
Oxygen desaturation index (>3% dips), events/h	2.8 (0.5 to 8.1)	18.5 (6.8 to 47.0)^a^	20.2 (5.0 to 44.5)^a^	3.3 (1.6 to 7.9)	8.1 (4.3 to 20.2)^a^^,^^b^	7.6 (3.3 to 14.6)^a^^,^^b^	−15.4 (−21.4 to −9.3)	−15.7 (−21.8 to −9.5)
Apnea/hypopnea index, events/h	20.5 (12.3 to 48.1)	39.4 (19.3 to 66.2)^a^	38.0 (15.6 to 63.2)^a^	25.9 (16.3 to 37.1)	24.7 (13.2 to 33.7)^b^	21.6 (13.4 to 38.7)^b^	−18.7 (−25.3 to −12.0)	−17.7 (−24.3 to −11.0)
Central apnea/hypopnea index, events/h	1.6 (0.3 to 2.8)	13.2 (3.1 to 27.7)^a^	11.1 (2.7 to 32.1)^a^	1.5 (0.5 to 2.5)	3.7 (2.0 to 8.3)^a^^,^^b^	4.2 (1.4 to 11.8)^a^^,^^b^	−12.0 (−18.5 to −5.5)	−12.1 (−18.7 to −5.5)
Obstructive apnea/hypopnea index, events/h	18.2 (11.1 to 38.2)	19.2 (9.3 to 35.8)	14.1 (8.6 to 36.1)	23.2 (15.6 to 36.0)	17.4 (7.1 to 24.8)^a^	15.6 (8.5 to 27.0)^a^	−6.7 (−11.5 to −1.8)	−5.5 (−10.4 to −0.6)
Periodic breathing, min	0 (0 to 0)	26 (4 to 70)^a^	22 (3 to 85)^a^	0 (0 to 0)	4 (0 to 15)^a^^,^^b^	4 (0 to 24)^a^^,^^b^	−40 (−63 to −16)	−41 (−64 to −17)
Cerebral tissue oxygen, %	70 (67 to 73)	67 (62 to 70)^a^	NA	68 (65 to 74)	67 (61 to 72)^a^	NA	1.0 (−2.0 to 4.0)	NA
Cerebral oxygen desaturation index, events/h	0.7 (0.1 to 2.0)	3.6 (0.6 to 11.4)^a^	NA	0.8 (0.1 to 1.9)	2.0 (0.4 to 4.9)^a^	NA	−4.5 (−0.9 to −8.1)	NA
Heart rate, bpm	65 (59 to 70)	69 (63 to 73)^a^	67 (60 to 72)^a^	64 (60 to 68)	69 (62 to 75)^a^	60 (54 to 67)^a^^,^^b^	2 (0 to 4)	−5 (−8 to −3)
Subjective sleep quality, %^c^	58 (43 to 82)	55 (30 to 71)	55 (40 to 69)	50 (38 to 73)	57 (39 to 75)	64 (45 to 78)^a^^,^^b^	11 (−1 to 22)	12 (0 to 23)
Sleep latency, min^c^	30 (10 to 45)	30 (10 to 55)	30 (10 to 30)	30 (10 to 30)	30 (10 to 60)	30 (10 to 60)	1 (−20 to 21)	−1 (−22 to 19)
Awakenings at night, No.^c^	2 (1 to 3)	2 (1 to 3)	2 (1 to 3)	2 (1 to 3)	2 (1 to 3)	2 (1 to 3)	0 (−1 to 1)	0 (−1 to 1)
Night-time spent awake, min^c^	13 (5 to 30)	10 (5 to 25)	10 (5 to 20)	5 (5 to 15)	15 (8 to 30)^a^	10 (5 to 30)^a^	22 (−2 to 47)	5 (−20 to 30)

Abbreviations: IQR, interquartile range; NA, not assessed; Spo_2_, arterial oxygen saturation measured by pulse oximetry.

^a^ *P* < .05 vs 760 m.

^b^ *P* < .05 between dexamethasone and placebo at the same corresponding altitude and day.

^c^ Assessed subjectively. Subjective sleep quality was assessed by a 100-mm visual analog scale ranging from 0 (extremely bad) to 100 (excellent).

The oxygen desaturation index increased in both groups with altitude ascent; however, dexamethasone mitigated this effect by 15.4 events/h (95% CI, 9.3-21.4) in the first night at 3100 m and by 17.7 events/h (95% CI, 9.5-21.8) in the second night at 3100 m, and it prevented an altitude-induced increase in the AHI (median for placebo in the first night at 3100 m: from 20.5 events/h [IQR, 12.3-48.1] to 39.4 events/h [IQR, 19.3-66.2]; dexamethasone: from 25.9 events/h [IQR, 16.3-37.1] to 24.7 events/h [IQR,13.2-33.7]). An increase in AHI was mainly prevented by a major increase in the central AHI and, to a lesser extent, in the obstructive AHI. Dexamethasone significantly reduced the nighttime spent with periodic breathing compared with placebo (median, 4 minutes [IQR, 0-15] vs 26 minutes [IQR, 4-70]). During the first night at 3100 m, patients receiving placebo developed a slight reduction in cerebral tissue oxygenation (median, from 70% [IQR, 67%-73%] to 67% [IQR, 62%-70%]) and an increase in the cerebral oxygen desaturation index (median, from 0.7 events/h [IQR, 0.1-2.0] to 3.6 events/h [IQR, 0.6-11.4]); similar changes were observed in the dexamethasone group (cerebral tissue oxygenation: median, from 68% [IQR, 65%-74%] to 67% [IQR, 61%-72%] and cerebral oxygen desaturation index: from 0.8 events/h [IQR, 0.1-1.9] to 2.0 events/h [IQR, 0.4-4.9]) (Table 2). At 3100 m on night 1, heart rate was increased in both groups compared with the rate at 760 m (median for placebo: from 65 bpm [IQR, 59-70] to 69 bpm [IQR, 63-73]; dexamethasone: from 64 bpm [IQR, 60-68] to 69 bpm (IQR, 62-75]) (Table 2).

Patients receiving dexamethasone perceived better sleep quality than patients receiving placebo (Table 2, Figure 2); mean differences with dexamethasone during nights 1 and 2 at 3100 m were 11% (95% CI, −1% to 22%) and 12% (95% CI, 0%-23%). There were no significant between-group differences in subjective estimates of sleep latency, nocturnal time spent awake, and awakenings. The psychomotor vigilance test reaction time and subjective sleepiness were similar at low and high altitude and no between-group difference was noted (Table 3).

**Table 3.** Daytime Assessments

Variable	Median (IQR)						Treatment Effect at 3100 m, Mean Difference (95% CI)	
	Placebo Group (n = 48)			Dexamethasone Group (n = 56)				
	760 m	3100 m		760 m	3100 m		Day 1	Day 2
		Day 1	Day 2		Day 1	Day 2		
Karolinska sleepiness score^a^	3 (3 to 5)	3 (3 to 5)^b^	3 (3 to 5)	3 (3 to 5)	3 (3 to 5)	3 (3 to 5)	−1 (−2 to 0)	0 (−1 to 0)
Reaction time, ms	344 (297 to 422)	327 (286 to 393)	NA	317 (279 to 408)	336 (287 to 401)	NA	38 (−28 to 104)	NA
Weight, kg	73.0 (65.6 to 80.9)	72.1 (65.7 to 79.2)	73.2 (66.0 to 80.2)	72.0 (63.0 to 80.0)	70.5 (62.6 to 80.0)	72.4 (63.3 to 80.7)	0 (−1 to 1)	0 (−1 to 0)
BP, mm Hg								
Systolic	131 (111 to 141)	128 (118 to 141)	134 (120 to 142)^b^	130 (112 to 142)	126 (113 to 134)	129 (120 to 141)	−5 (−10 to 0)	−3 (−9 to 3)
Diastolic	82 (73 to 89)	82 (74 to 86)	82 (75 to 89)	79 (73 to 87)	77 (69 to 84)^b^^,^^c^	81 (74 to 85)	−5 (−9 to −1)	−4 (−7 to 0)
Median (IQR) of the mean	98 (87 to 105)	96 (89 to 104)	101 (91 to 107)^b^	95 (87 to 107)	91 (86 to 101)^b^^,^^c^	97 (91 to 104)	−5 (−9 to −1)	−3 (−7 to 0)
Heart rate, bpm	64 (59 to 70)	68 (62 to 73)^b^	66 (60 to 73)	63 (58 to 70)	67 (60 to 71)^b^	59 (55 to 69)^c^	0 (−3 to 3)	−4 (−7 to −1)
Arterial pH	7.39 (7.38 to 7.41)	7.42 (7.41 to 7.44)^b^	NA	7.40 (7.39 to 7.42)	7.43 (7.42 to 7.45)^b^^,^^c^	NA	0.01 (0.00 to 0.02)	NA
Paco_2_, kPa	5.1 (4.8 to 5.4)	4.6 (4.4 to 4.9)^b^	NA	5.2 (4.8 to 5.4)	4.4 (4.1 to 4.7)^b^^,^^c^	NA	−0.2 (−0.4 to −0.1)	NA
Pao_2_, kPa	10.0 (9.2 to 10.7)	8.0 (7.8 to 8.4)^b^	NA	9.6 (9.2 to 10.0)	8.2 (7.9 to 8.7)^b^	NA	0.5 (0.1 to 0.9)	NA
Sao_2_, %	95 (93 to 95)	89 (88 to 90)^b^	NA	94 (93 to 95)	90 (89 to 91)^b^^,^^c^	NA	0.01 (0.00 to 0.02)	NA
DAaPo_2_, kPa	3.1 (2.5 to 4.0)	1.1 (0.5 to 1.5)^b^	NA	3.6 (3.0 to 3.9)	1.1 (0.6 to 1.4)^b^	NA	−0.2 (−0.6 to 0.2)	NA
HCO_3_, mEq/L	23.3 (21.7 to 24.4)	22.1 (20.8 to 23.0)^b^	NA	23.2 (22.0 to 24.4)	21.4 (20.6 to 22.8)^b^	NA	−0.6 (−1.4 to 0.1)	NA
Hematocrit, %	44 (40 to 46)	44 (41 to 46)	NA	44 (41 to 46)	44 (41 to 47)^b^	NA	0 (0 to 0)	NA
Hemoglobin, g/dL	14.8 (13.5 to 15.7)	14.9 (13.8 to 15.6)	NA	15.0 (13.8 to 15.8)	14.9 (14.1 to 16.1)^b^	NA	0.1 (−0.1 to 0.3)	NA
Glucose, mg/dL	126.1 (108.1 to 144.1)	133.3 (109.9 to 183.2)	NA	120.7 (106.3 to 142.3)	167.6 (140.5 to 205.4)^b^^,^^c^	NA	52.3 (30.6 to 73.9)	NA
FEV_1_, L	2.7 (2.3 to 3.0)	2.6 (2.3 to 3.0)	2.6 (2.1 to 2.9)	2.5 (1.9 to 2.9)	2.6 (1.9 to 2.9)	2.5 (2.0 to 2.9)	0.1 (0.0 to 0.2)	0.1 (0.0 to 0.2)
% Predicted	96 (77 to 109)	93 (73 to 105)	90 (79 to 105)	85 (73 to 102)	87 (74 to 103)	90 (70 to 100)	3 (−1 to 6)	2 (−2 to 5)
FVC, L	4.3 (3.7 to 4.9)	4.1 (3.5 to 4.8)	4.1 (3.5 to 4.8)^b^	4.1 (3.3 to 4.5)	4.2 (3.5 to 4.5)	4.0 (3.3 to 4.6)	0.1 (−0.1 to 0.2)	0 (−0.1 to 0.1)
% Predicted	122 (104 to 137)	118 (99 to 136)	121 (102 to 129)	115 (98 to 125)	114 (98 to 125)	115 (97 to 126)	2 (−2 to 5)	0 (−3 to 4)
FEV_1_/FVC	0.65 (0.60 to 0.68)	0.65 (0.60 to 0.68)	0.65 (0.59 to 0.68)	0.63 (0.56 to 0.66)	0.63 (0.60 to 0.68)^b^	0.63 (0.58 to 0.68)	0.01 (−0.01 to 0.03)	0.01 (0.00 to 0.03)

Abbreviations: BP, blood pressure; DAaPo_2_, alveolar-arterial Po_2_ difference^23^; FEV_1_, forced expiratory volume in the first second of expiration; FVC, forced vital capacity; HCO_3_, bicarbonate concentration; IQR, interquartile range; NA, not assessed; Paco_2_, partial pressure of carbon dioxide; Pao_2_, partial pressure of oxygen; Sao_2_, arterial oxygen saturation.

SI conversion factors: to convert glucose to millimoles per liter, multiply by 0.0555; HCO_3_ to millimoles per liter, multiply by 1; hematocrit to proportion of 1.0, multiply by 0.01; hemoglobin to grams per liter, multiply by 10.

^a^ Karolinska sleepiness score ranges from 1 (extremely alert) to 9 (very sleepy, great effort to keep alert, fighting sleep).

^b^ *P* < .05 vs 760 m.

^c^ *P* < .05 between dexamethasone and placebo at the corresponding altitude and day.

A significant increase in mean arterial blood pressure in the placebo group after night 2 at 3100 m by 3 mm Hg (95% CI, 1-6 mm Hg), and a significant blood pressure–reducing effect of dexamethasone at the higher altitude were observed after night 1 at 3100 m by −5 mm Hg (95% CI, −9 to −1 mm Hg); after night 2 at 3100 m by −3 mm Hg (95% CI, −7 to 0 mm Hg) (Table 3; eFigure in Supplement 2). Arterial blood gas analyses revealed altitude-induced hypocapnia and an increase in pH that was more pronounced in patients receiving dexamethasone who had less altitude-induced hypoxemia than patients receiving placebo. Spirometry findings did not change over the course of the study. Patients assigned to dexamethasone vs placebo had a higher blood glucose concentration (median, 167.6 mg/dL [IQR, 140.5-205.4] vs 133.3 mg/dL [IQR, 109.9-183.2] [to convert to millimoles per liter, multiply by 0.0555]) (Table 3), and 16 of them (27.6%) had asymptomatic hyperglycemia (glucose level ≥200 mg/dL).

In regression analysis, lower Pao_2_ (coefficient, 0.50; 95% CI, 0.17-0.84) and FEV_1 _(coefficient, 0.02; 95% CI, 0.00-0.04), and a higher Paco_2_ (coefficient, −1.15; 95% CI, −1.89 to −0.41) and AHI (coefficient, −0.03; 95% CI, −0.05 to −0.01) at 760 m were associated with lower mean nocturnal Spo_2_ at 3100 m (eTable 2 in Supplement 2). Furthermore, the risk of experiencing an adverse event that required an intervention (eg, oxygen or drug therapy) and premature study termination was increased by a mean of 3.84 (95% CI, 1.28-11.11) per each 1-kPa reduction in Pao_2_ at 760 m, and this risk was reduced 20 times (95% CI, 4.34 to >100) by preventive dexamethasone treatment (eTable 3 in Supplement 2). In contrast to Pao_2_, daytime Spo_2_ level at 760 m was not a significant predictor of premature study termination in the corresponding regression model (eTable 4 in Supplement 2).

## Discussion

This randomized, placebo-controlled, double-blind trial in lowlanders in Central Asia with mild to moderate COPD (GOLD grade 1-2) demonstrates that preventive dexamethasone treatment mitigated the altitude-induced decrease in the nocturnal Spo_2_ and prevented an increase in AHI during 2 nights at 3100 m. These favorable effects were associated with improvements in subjective sleep quality. Therefore, dexamethasone may be used in selected patients with COPD undergoing high altitude travel to prevent severe nocturnal hypoxemia.

In healthy lowlanders, an altitude-dependent reduction in nocturnal Spo_2_, emergence of periodic breathing, sleep disturbances, and impaired cognitive performance have been reported.^2^ For example, in 51 healthy lowlanders (median age, 24 years), the median nocturnal Spo_2_ in the night after ascent from 490 to 2590 m was 90%, and the AHI was 13.1 events/h.^24^ In 40 healthy volunteers (mean age, 40 years) ascending from 555 to 3150 m, the mean Spo_2_ during the first night at high altitude was 83%, and the AHI was 7.4 events/h.^25^

In patients with COPD, effects of altitude travel have not been extensively studied. In a study of 32 lowlanders with COPD (median FEV_1_ 59% predicted) ascending from 490 to 2590 m, a reduction was observed in the median nocturnal Spo_2_ from 92% to 85% and emergence of predominantly central sleep apnea (increase in median AHI from 15.4 to 55.7 events/h).^5^ This degree of nocturnal hypoxemia and central sleep apnea was similar to that in patients in the present study with less severe COPD (placebo group, Table 2) exposed to a higher altitude (3100 m). Despite a degree of hypoxemia in the patients with COPD in the present study similar to that of healthy individuals studied at 3150 m,^25^ the AHI of patients with COPD was higher (ie, 39.4 events/h in the present study during night 1 at 3100 m with placebo vs 7.4 events/h at 3150 m). Reduced stability in the control of breathing of patients with COPD compared with healthy individuals may be due to their older age and/or lung disease, which might have promoted central apnea by an increased ventilatory drive.^26^ Because we did not measure ventilatory drive and arterial blood gas analyses are not available from the previous investigation,^25^ we are unable to corroborate this hypothesis. The baseline AHI values at 760 m of 20.5 and 25.9 events/h in participants of the present study are within the IQR of values reported in a sample of the general population of individuals older than 40 years (ie, 7.2-27.1 events/h).^27^ In the absence of symptoms, we have no evidence that the participants in the present study experienced obstructive sleep apnea syndrome—a condition known to predispose to exacerbated sleep apnea during altitude sojourns.^3^

To our knowledge, the present trial is the first to evaluate prevention of altitude-related nocturnal hypoxemia and breathing disturbances in patients with COPD. We selected dexamethasone for this trial because it has been shown to prevent AMS in healthy individuals^8,9^; furthermore, glucocorticoids are used to treat COPD exacerbations by decreasing airway inflammation and airflow obstruction.^11^ Moreover, in otherwise healthy individuals susceptible to high-altitude pulmonary edema ascending rapidly to 4559 m, dexamethasone improved nocturnal oxygenation and reduced high-altitude periodic breathing in addition to reducing pulmonary artery pressure.^10,28^ The reduction in high-altitude periodic breathing was associated with a reduced eupneic end-tidal Pco_2_ (the surrogate of Paco_2_). As the apnea threshold was also reduced, the CO_2_ reserve (the difference between eupneic Paco_2_ and apnea threshold of Paco_2_), one of the determinants of breathing stability, was maintained. In the present study, patients with COPD receiving dexamethasone had greater altitude-related hypocapnia than patients receiving placebo. Assuming no change in the CO_2_ reserve, the lower AHI at 3100 m in patients receiving dexamethasone might therefore be associated with a greater ventilatory overshoot required to cross the apnea threshold because of the hyperbolic shape of the alveolar ventilation vs Paco_2_ relationship.^17,29^ A reduced hypoxic ventilatory drive owing to the higher Pao_2_ in patients receiving dexamethasone might have additionally stabilized their control of breathing. There is increasing evidence that pulmonary hypertension is associated with breathing instability.^30,31^ Reducing hypoxic pulmonary vasoconstriction by the dexamethasone-mediated increase in alveolar ventilation and alveolar Po_2_ might have contributed to the reduced AHI level.

The relevance of the improvement in hypoxemia by dexamethasone is uncertain as the minimal clinically important degree of alleviation of hypoxemia is unknown and symptoms of AMS were not reduced.^12^ However, it is generally accepted that the health risks of hypoxemia increase with its severity, in particular, in patients with cardiopulmonary disease, such as the participants in the present study. Air travel recommendations suggest that patients with stable respiratory disease reaching a Spo_2_ lower than 85% during a normobaric hypoxic challenge test (breathing 15% fractional inspired oxygen for 20 minutes) use in-flight oxygen.^22^ For comparison, in the present study, preventive dexamethasone treatment reduced the proportion of participants with mean nocturnal Spo_2_ below 85% by more than half compared with placebo (25.0% vs 55.2%, *P* = .001).

In exploratory regression analyses, a low Pao_2_ (but associated with Spo_2 _≥92% according to inclusion criteria) and higher values of Paco_2_ and AHI at 760 m were associated with more severe hypoxemia at 3100 m—a risk that was reduced by dexamethasone (eTable 2 in Supplement 2). Moreover, the risk of experiencing an adverse event requiring study termination according to safety rules was increased nearly 4 times per each 1-kPa reduction in Pao_2_ at 760 m, but the risk was reduced 20 times by preventive dexamethasone treatment (eTable 3 in Supplement 2).

The improvement of subjective sleep quality with dexamethasone during night 2 at 3100 m (12 mm on the visual analog scale) was similar to the difference of 10 mm found to be clinically important in patients with insomnia.^32^ Subjective sleepiness and psychomotor vigilance test reaction time remained unchanged with ascent to a higher altitude (Table 3). It is uncertain whether the mild altitude-induced reduction in cerebral tissue oxygenation detected by near-infrared spectroscopy was not strong enough to cause measurable effects in these outcomes in both patient groups.

A total of 16 (27.6%) of the patients with COPD assigned to dexamethasone had drug-induced hyperglycemia.^33^ No other relevant adverse effects of the short-term use of dexamethasone were noted in the present study.

### Limitations

Limitations of the present trial include the population with predominantly mild COPD during 2 nights at altitude. The results can therefore not be extrapolated to patients with more severe COPD and longer altitude sojourns. Because this study was performed in residents of Central Asia it is uncertain whether the results apply to people in other regions of the world. Nevertheless, our analyses suggest that severe hypoxemia assessed by the Pao_2_ at low altitude (but not by pulse oximetry, which may overestimate arterial oxygen saturation in smokers with elevated carboxyhemoglobin levels), higher levels of Paco_2_, and sleep apnea may help to identify patients with more severe COPD at risk of experiencing adverse health effects at high altitudes (eTables 2-4 in Supplement 2).

## Conclusions

The results of the present randomized clinical trial reveal that lowlanders with mild to moderate COPD traveling to high altitude experienced nocturnal hypoxemia and central sleep apnea. Preventive treatment with dexamethasone reduced the risk of severe hypoxemia and/or adverse events requiring premature descent to lower altitude. Because dexamethasone predisposes to hyperglycemia, its use for prevention of altitude-related hypoxemia, as well as breathing and sleep disturbances in patients with COPD, should be limited. We suggest that a short course of preventive dexamethasone treatment be considered only in patients with COPD who do not have diabetes or uncontrolled cardiovascular disease and are not heavy smokers. In addition, treatment should be considered if the evaluation before ascent shows moderate hypoxemia (reduced Pao_2_ but Spo_2 _≥92%), and a Paco_2_ value that is in the high normal range, and oxygen therapy during high-altitude travel is not feasible.

## Notes

Supplement 1. **Study Protocol**

Supplement 2. **eMethods.** Sleep Study Analysis and Additional Measurements **eTable 1.** Altitude-Induced Changes in Selected Variables **eTable 2.** Multivariable Regression Analysis of Predictors of Mean Nocturnal Oxygen Saturation (Spo_2_) at 3100 m **eTable 3.** Logistic Regression Analysis of Predictors (Including PaO_2_) of Premature Study Termination at 3100 m Due to Adverse Events **eTable 4.** Logistic Regression Analysis of Predictors (Including Spo_2_) of Early Study Termination at 3100 m Due to Adverse Events **eFigure.** Effect of Altitude and Dexamethasone on Apneas/Hypopneas and Blood Pressure **eReferences**

Supplement 3. **Data Sharing Statement**

## References

[1] Tourism statistics—intra-EU tourism flows. http://ec.europa.eu/eurostat/statistics-explained/index.php/Tourism_statistics_-_intra-EU_tourism_flows. Updated May 2018. Accessed February 5, 2018.

[2] Bloch KE, Buenzli JC, Latshang TD, Ulrich S Sleep at high altitude: guesses and facts. J Appl Physiol (1985). 2015;119(12):-. doi:10.1152/japplphysiol.00448.2015 26229000

[3] Nussbaumer-Ochsner Y, Schuepfer N, Ulrich S, Bloch KE Exacerbation of sleep apnoea by frequent central events in patients with the obstructive sleep apnoea syndrome at altitude: a randomised trial. Thorax. 2010;65(5):429-435. doi:10.1136/thx.2009.125849 20435865

[4] Furian M, Hartmann SE, Latshang TD, Exercise performance of lowlanders with COPD at 2,590 m: data from a randomized trial. Respiration. 2018;95(6):422-432. doi:10.1159/000486450 29502125

[5] Latshang TD, Tardent RPM, Furian M, Sleep and breathing disturbances in patients with chronic obstructive pulmonary disease traveling to altitude: a randomized trial [published online December 4, 2018]. Sleep. doi:10.1093/sleep/zsy203 30517695

[6] Latshang TD, Nussbaumer-Ochsner Y, Henn RM, Effect of acetazolamide and autoCPAP therapy on breathing disturbances among patients with obstructive sleep apnea syndrome who travel to altitude: a randomized controlled trial. JAMA. 2012;308(22):2390-2398. doi:10.1001/jama.2012.94847 23232895

[7] Adamson R, Swenson ER Acetazolamide use in severe chronic obstructive pulmonary disease. pros and cons. Ann Am Thorac Soc. 2017;14(7):1086-1093. 28622013 10.1513/AnnalsATS.201701-016FR

[8] Montgomery AB, Luce JM, Michael P, Mills J, Mills J Effects of dexamethasone on the incidence of acute mountain sickness at two intermediate altitudes. JAMA. 1989;261(5):734-736. doi:10.1001/jama.1989.03420050084045 2911170

[9] Zheng CR, Chen GZ, Yu J, Inhaled budesonide and oral dexamethasone prevent acute mountain sickness. Am J Med. 2014;127(10):1001-1009.e2. doi:10.1016/j.amjmed.2014.04.012 24784698

[10] Maggiorini M, Brunner-La Rocca HP, Peth S, Both tadalafil and dexamethasone may reduce the incidence of high-altitude pulmonary edema: a randomized trial. Ann Intern Med. 2006;145(7):497-506. doi:10.7326/0003-4819-145-7-200610030-00007 17015867

[11] Global Initiative for Chronic Obstructive Lung Disease. Global strategy for the diagnosis; management and prevention of COPD (GOLD). https://goldcopd.org. Accessed July 31, 2018.

[12] Furian M, Lichtblau M, Aeschbacher SS, Efficacy of dexamethasone in preventing acute mountain sickness in COPD patients: randomized trial. Chest. 2018;154(4):788-797. doi:10.1016/j.chest.2018.06.006 29909285

[13] Muralt L, Furian M, Lichtblau M, Postural control in lowlanders with COPD traveling to 3100 m: data from a randomized trial evaluating the effect of preventive dexamethasone treatment. Front Physiol. 2018;9:752. doi:10.3389/fphys.2018.00752 29988503 PMC6024910

[14] Schulz KF, Altman DG, Moher D, Group C; CONSORT Group CONSORT 2010 statement: updated guidelines for reporting parallel group randomised trials. BMJ. 2010;340:c332. doi:10.1136/bmj.c332 20332509 PMC2844940

[15] Jones PW, Harding G, Berry P, Wiklund I, Chen WH, Kline Leidy N Development and first validation of the COPD Assessment Test. Eur Respir J. 2009;34(3):648-654. doi:10.1183/09031936.00102509 19720809

[16] Ulrich S, Nussbaumer-Ochsner Y, Vasic I, Cerebral oxygenation in patients with OSA: effects of hypoxia at altitude and impact of acetazolamide. Chest. 2014;146(2):299-308. doi:10.1378/chest.13-2967 24811331

[17] Nussbaumer-Ochsner Y, Ursprung J, Siebenmann C, Maggiorini M, Bloch KE Effect of short-term acclimatization to high altitude on sleep and nocturnal breathing. Sleep. 2012;35(3):419-423. doi:10.5665/sleep.1708 22379248 PMC3274343

[18] Kaida K, Takahashi M, Åkerstedt T, Validation of the Karolinska sleepiness scale against performance and EEG variables. Clin Neurophysiol. 2006;117(7):1574-1581. doi:10.1016/j.clinph.2006.03.011 16679057

[19] Quanjer PH, Stanojevic S, Cole TJ, ; ERS Global Lung Function Initiative Multi-ethnic reference values for spirometry for the 3-95-yr age range: the global lung function 2012 equations. Eur Respir J. 2012;40(6):1324-1343. doi:10.1183/09031936.00080312 22743675 PMC3786581

[20] Basner M, Dinges DF Maximizing sensitivity of the psychomotor vigilance test (PVT) to sleep loss. Sleep. 2011;34(5):581-591. doi:10.1093/sleep/34.5.581 21532951 PMC3079937

[21] White IR, Royston P, Wood AM Multiple imputation using chained equations: issues and guidance for practice. Stat Med. 2011;30(4):377-399. doi:10.1002/sim.4067 21225900

[22] Shrikrishna D, Coker RK; Air Travel Working Party of the British Thoracic Society Standards of Care Committee Managing passengers with stable respiratory disease planning air travel: British Thoracic Society recommendations. Thorax. 2011;66(9):831-833. doi:10.1136/thoraxjnl-2011-200694 21807654

[23] Crapo RO, Jensen RL, Hegewald M, Tashkin DP Arterial blood gas reference values for sea level and an altitude of 1,400 meters. Am J Respir Crit Care Med. 1999;160(5, pt 1):1525-1531. doi:10.1164/ajrccm.160.5.9806006 10556115

[24] Latshang TD, Lo Cascio CM, Stöwhas AC, Are nocturnal breathing, sleep, and cognitive performance impaired at moderate altitude (1,630-2,590 m)? Sleep. 2013;36(12):1969-1976. doi:10.5665/sleep.3242 24293773 PMC3825448

[25] Tseng C-H, Lin F-C, Chao H-S, Tsai H-C, Shiao G-M, Chang S-C Impact of rapid ascent to high altitude on sleep. Sleep Breath. 2015;19(3):819-826. doi:10.1007/s11325-014-1093-7 25491080

[26] Luo YM, He BT, Wu YX, Neural respiratory drive and ventilation in patients with chronic obstructive pulmonary disease during sleep. Am J Respir Crit Care Med. 2014;190(2):227-229. doi:10.1164/rccm.201402-0302LE 25025355 PMC4226055

[27] Heinzer R, Vat S, Marques-Vidal P, Prevalence of sleep-disordered breathing in the general population: the HypnoLaus study. Lancet Respir Med. 2015;3(4):310-318. doi:10.1016/S2213-2600(15)00043-0 25682233 PMC4404207

[28] Nussbaumer-Ochsner Y, Schuepfer N, Ursprung J, Siebenmann C, Maggiorini M, Bloch KE Sleep and breathing in high altitude pulmonary edema susceptible subjects at 4,559 meters. Sleep. 2012;35(10):1413-1421. doi:10.5665/sleep.2126 23024440 PMC3443768

[29] Dempsey JA, Smith CA, Przybylowski T, The ventilatory responsiveness to CO_2_ below eupnoea as a determinant of ventilatory stability in sleep. J Physiol. 2004;560(pt 1):1-11. doi:10.1113/jphysiol.2004.072371 15284345 PMC1665213

[30] Latshang TD, Furian M, Aeschbacher SS, Association between sleep apnoea and pulmonary hypertension in Kyrgyz highlanders. Eur Respir J. 2017;49(2):1-10. doi:10.1183/13993003.01530-2016 28007792

[31] Thurnheer R, Ulrich S, Bloch KE Precapillary pulmonary hypertension and sleep-disordered breathing: is there a link? Respiration. 2017;93(1):65-77. doi:10.1159/000452957 27884004

[32] Zisapel N, Nir T Determination of the minimal clinically significant difference on a patient visual analog sleep quality scale. J Sleep Res. 2003;12(4):291-298. doi:10.1046/j.0962-1105.2003.00365.x 14633240

[33] International Diabetes Federation Guideline Development Group Global guideline for type 2 diabetes. Diabetes Res Clin Pract. 2014;104(1):1-52. doi:10.1016/j.diabres.2012.10.001 24508150

