# Supplementary material for: Effect of Dexamethasone on Nocturnal Oxygenation in Lowlanders With Chronic Obstructive Pulmonary Disease Traveling to 3100 Meters: A Randomized Clinical Trial
Source: JAMA Netw Open. 2019 Feb 22;2(2):e190067. doi: 10.1001/jamanetworkopen.2019.0067 (PMC6484579; doi:10.1001/jamanetworkopen.2019.0067)
Supplement: Supplement 1. — Study Protocol [file jamanetwopen-2-e190067-s001.pdf]

Prevention of Acute Mountain Sickness in Patients with Chronic Obstructive Pulmonary Disease

Randomized, Placebo-controlled Double-blind Trial Evaluating Effectiveness of Preventive Dexamethasone Therapy

Joint Swiss-Kyrgyz Research Project

Principal investigator and correspondence

Prof. Dr. Konrad E. Bloch, Klinik für Pneumologie, Bereich HGT, UniversitätsSpital Zürich

Rämistr. 100, 8091 Zürich. Tel.: 044 255 38 28, Fax.: 044 255 44 51

Email: konrad.bloch@usz.ch

Swiss Investigators

Prof. Konrad E. Bloch, MD, PD Silvia Ulrich, MD, Tsogyal D. Latshang, MD

Klinik für Pneumologie, Bereich Herz-Gefäss-Thorax, UniversitätsSpital Zürich

Kyrgyz Investigators

Prof. Talant Sooronbaev, MD

National Center for Cardiology and Internal Medicine, Bishkek, Kyrgyzstan.

Academician Prof. Almaz Aldashev, PhD

Research Institute for Molecular Biology, Kyrgyz State University, Bishkek, Kyrgyzstan

1. Summary

Background and Purpose: COPD is one of the leading causes of mortality, morbidity and health care costs worldwide. The disease is characterized by chronic airflow obstruction related to airway inflammation, remodeling and parenchymal destruction of the lung. Many patients with COPD desire to travel to altitude but recent studies performed at our center have shown that COPD patients experience exacerbated dyspnea, reduced exercise performance, and disturbed sleep when staying at even moderate altitude (Davos, 2590 m). Whether drugs that prevent altitude related illness in climbers to very high altitude are effective in COPD patients who experience a similar degree of hypoxemia already at lower altitude has not been studied. As such research might open new opportunities to prevent adverse health effects related to hypobaric hypoxia in COPD patients we have designed the current study. Its purpose is to evaluate the effectiveness of dexamethasone, a drug used by mountaineers to prevent acute mountain sickness (AMS), in preventing AMS and other adverse health effects of altitude in COPD patients. As we have a very fruitful cooperation with pulmonary physicians at the Kyrgyz State University in Bishkek, Kyrgyzstan, we plan to perform the trial in this central Asian country with a high prevalence of COPD and many settlements at high altitude.

Main hypothesis: In Kyrgyz lowlanders with COPD travelling to high altitude prophylactic dexamethasone treatment reduces the incidence of AMS.

Experimental design and methods: This will be a randomized, placebo-controlled, double-blind parallel trial evaluating the efficacy of dexamethasone prophylaxis in reducing the incidence of AMS in lowlanders with COPD (GOLD grade 1-2) travelling to altitude. Participants living in Bishkek, Kyrgyzstan (700m), will be transferred by car within 4h to the Tuja Ashu high altitude clinic (3200 m), and stay there for 2 days. Dexamethasone 2x4mg/day (or placebo), will be administered on the day of departure from 700 m and during the stay at altitude. Outcomes will be assessed at 700 m and during the stay at 3200 m. Findings in patients using dexamethasone and placebo will be compared. The main outcome will be the cumulative incidence of AMS during the stay at 3200 m. To detect a 50% reduction in AMS incidence by dexamethasone compared to placebo, 100 participants will be recruited (alpha 0.05, 80% power, 15% drop-out rate).

Expected value of the project: This randomized trial will provide a long-needed scientific basis for counselling COPD patients planning altitude travel. Although oxygen supplementation may be a means to prevent altitude related illness it requires heavy equipment (either pressurized bottles or an oxygen concentrator) and it is therefore inconvenient or not feasible during altitude travel. Demonstrating effectiveness of a drug like dexamethasone in preventing altitude illness would therefore be highly desirable for a large number of COPD patients going to mountain areas or travelling by airplane. Performing the study in Kyrgyzstan has some advantages over performing it in Switzerland as it offers cost savings. Our Kyrgyz partners expect that an adequate number of Kyrgyz COPD patients are keen to participate in the proposed study. Taking an active part in this clinical trial will give the opportunity to young Kyrgyz clinicians and researchers to acquire new knowledge and skills.

2. Background

Health risks and preventive measures for COPD patients travelling to altitude

In a systematic search of the literature we have identified very few and only observational studies on the potential hazards of altitude in patients with COPD. We refer to a previous grant application to the Lunge Zurich for a detailed review. The lack of robust evidence in this filed has prompted us to undergo a series of systematic investigations.

In a first randomized cross-over study we studied 40 patients with COPD (Gold grade 2-3) in Zürich, in Davos Clavadel (1650 m) and on the Jakobshorn (2590 m) during 2 days each. The main outcome was the 6 min walk distance which was significantly reduced at 1650 and 2590 m compared to 490 m.[1] Our physiologic studies revealed that the exercise limitation was related to a reduced oxygen availability in the brain and in skeletal muscles but not to mechanical ventilatory limitation. [2] Another finding of the cited study was that COPD patients had pronounced nocturnal hypoxemia, periodic breathing and sleep disturbances.[3] Moreover, patients experienced systemic and pulmonary hypertension with right and left ventricular dysfunction at 2590 m [4] and a prolongation of the QTc interval measured in the ECG as a surrogate of the risk of malignant arrhythmia. The described findings are currently in preparation for publication and have been received with interest international meetings.

Treatment of COPD patients at altitude

In ongoing studies performed in summer 2014 in Zurich and St. Moritz Salastrains (2044 m) we have evaluated the effect of nocturnal oxygen supplementation in comparison to sham oxygen (ambient air administration) on the exercise performance, nocturnal sleep and breathing, right and left ventricular function assessed by echocardiography, and several other physiologic outcomes. The findings of this randomized placebo-controlled trial are not yet available as the investigators are still blinded and more patients will be included during extension of the study. We found that recruiting COPD patients for this trial was not easy. Although already 33 patients have been studied in 2014, the goal of >40 participants has not been achieved yet.

Although oxygen supplementation may be a effective means to prevent altitude related illness it requires heavy equipment (either pressurized bottles or an oxygen concentrator) and it is therefore inconvenient or not feasible during altitude travel for some COPD patients. Demonstrating effectiveness of a drug in preventing altitude illness would therefore be highly desirable for a large number of COPD patients going to mountain areas or travelling by airplane.

Acetazolamide, a carbonic anhydrase inhibitor, acts as a respiratory stimulant by promoting renal bicarbonate excretion thus correcting the respiratory alkalosis induced by hypoxia. Theoretically, COPD patients travelling to altitude might benefit from acetazolamide as we have shown in patients with obstructive sleep apnea syndrome[5,6]. However, there is concern that combined pharmacologic and hypoxic stimulation of ventilation at altitude may exacerbate dyspnea, and even lead to respiratory muscle failure in COPD patients.

Dexamethasone, a potent drug for prevention and treatment of acute mountain sickness reduces the excessive rise in pulmonary artery pressure, improves exercise capacity and prevents high altitude pulmonary edema in susceptible subjects [7,8]. Glucocorticosteroids also improve airway inflammation and airflow obstruction in many COPD patients,

especially during exacerbations [9]. Whether COPD patients may benefit from preventive glucocorticoid treatment during an altitude sojourn has not been studied.

The purpose of the current project is therefore to evaluate effectiveness of dexametheasone treatment for prevention of adverse health effects in COPD patients at altitude. We decided to perform the study in cooperation with Prof. T. Sooronbaev and Prof. A. Aldashev at the Kyrgyz State University in Bishkek, Kyrgyzstan, since we have a favorable experience in cooperating with these colleages and as there are some advantages over performing the study in Kyrgyzstan as compared to Switzerland. Apart from cost savings we expect that our Kyrgyz partners will be able to recruit an adequate number of Kyrgyz COPD patients within a short time that allows us to complete the study within a short time, i.e., from May to August 2015. In addition to these aspects, the projects offers to the opportunity for young Kyrgyz clinicians and researchers who have no easy access to measurement techniques and research to acquire valuable knowledge and skills in good clinical practice while participating in this clinical trial.

3. Detailed research plan

Purpose and Hypotheses

The purpose of the current project is to evaluate in lowlanders with COPD whether the incidence of acute mountain sickness (AMS) is improved by prophylactic dexamethasone therapy.

Hypotheses

In Kyrgyz lowlanders with COPD ascending rapidly to 3200 m, prophylactic dexamethasone treatment reduces the incidence and severity of AMS during the stay at altitude.

Methods

Patients

Kyrgyz lowlanders with COPD living in the Bishkek area (mean altitude 700 m) will be recruited among patients of the outpatient clinic of the National Center for Cardiology and Internal Medicine in Bishkek.

Inclusion criteria

- COPD diagnosed according to GOLD criteria, grades 1-2, FEV1/FVC <0.7 and FEV1 >50 % predicted.
- Age 20 to 75 years, both genders
- Born, raised and currently living at low altitude (<800m)
- informed consent.

Exclusion criteria

- COPD exacerbation, severe COPD (i.e. GOLD 3, FEV1≤50% predicted), gas exchange impairment at 700 m (oxygen saturation on room air <92%).
- Diabetes, uncontrolled cardiovascular disease such as systemic arterial hypertension, coronary artery disease; previous stroke; OSA; pneumothorax in the last 2 months, untreated or symptomatic peptic ulcer disease, glaucoma.
- Internal, neurologic or psychiatric disease that interfere with protocol compliance including current heavy smoking (>20 cigarettes per day).

MEASUREMENTS AND INTERVENTIONS

HISTORY, QUESTIONNAIRE EVALUATION, CLINICAL EXAMINATION

A complete medical history will be obtained. Physical examination will include weight, height, blood pressure, pulse rate, cardiac and pulmonary auscultation. The NYHA functional class, the COPD assessment test (CAT) will be obtained[10]. Perceived exertion will be assessed by the Borg CR10 scale [11], dyspnea by the modified MRC dyspnea score [12], acute and chronic mountain sickness by the environmental symptoms questionnaire (AMS-c),[13] the Lake Louise AMS questionnaire, and the Quinghai CMS score, sleepiness will be assessed by the Epworth sleepiness scale[14] and the Karolinska and Stanford questionnaires, subjective sleep quality will be assessed by a visual analog scale. Postural control will be evaluated by letting patients stand on a balance platform the center of pressure path length will be recorded as described previously.[15] [16] The current medical treatment of patients will be noted and maintained during the study in the same dosage if feasible (including inhaled corticosteroids).[17]

EXERCISE PERFORMANCE, PULMONARY FUNCTION, AND BLOOD ANALYSIS

The 6 min walk test (6MWT) will be performed [18]. Patients will be monitored by pulse oximetry and fill in a 10 point Borg scale at the end of the test. Walk distance, SpO_2, pulse rate and blood pressure at beginning and end of the test will be recorded. Spirometry and nasal sniff inspiratory pressure measurements will be is performed.[19] Blood gas analysis of a radial artery sample will be obtained. Venous blood will be withdrawn to allow measurement of glucose, brain natriuretic peptide (NT-Pro-BNP) as a measure of heart failure, and to perform further biochemical studies.

ECHOCARDIOGRAPHY AND ELECTROCARDIOGRAM

Doppler-echocardiography and a 12 lead ECG will be performed as previously described. Systolic pulmonary artery pressure and right ventricular dimensions will be obtained,[20] mean pulmonary artery pressure will be estimated from acceleration time as previously described in Kyrgyz highlanders.

CARDIORESPIRATORY SLEEP AND PHYSIOLOGIC MONITORING

Continuous nocturnal measurements by portable devices (Alice PDX, Philips Respironics) will include nasal pressure swings, chest wall excursion, ECG, pulse oximetry and cerebral tissue oxygen saturation by non-invasive near-infrared spectroscopy by optical sensors placed on the scalp of the forehead.[21] A transcranial ultrasound probe will be placed on the scalp in the temporal region to monitor flow velocity in the media cerebral artery. Mean oxygen saturation and percent time with SpO_2 <90%, and <80%, will be computed. The apnea/hypopnea index (AHI) and oxygen desaturation index (SpO_2 dips >3%) will be computed as mean number of events/h.[22]

TREATMENT WITH DEXAMETHASONE, RANDOMIZATION AND BLINDING

COPD patients will receive dexamethasone tablets 4mg, twice a day (or an equally looking placebo), with breakfast and dinner. Treatment will start on the day before departure to altitude and will be continued throughout the altitude sojourn. Randomization and blinding will be achieved by letting participants draw a box containing the study drugs. An independent pharmacist will prepare and dispense identically looking active and placebo capsules in a way that provides a 1:1 balanced design. The dose of dexamethasone of 4 mg twice a day has been selected because it is the recommended dose to prevent and treat high altitude illness.[23] A dose of 8 mg of dexamethasone per day is equivalent to prednisone 53 mg, i.e., similar to the dose of 40 mg recommended for treatment of COPD exacerbations.[31] Patients will remain on their usual medication during the study including on bronchodilators, inhaled corticosteroids and other drugs.

STUDY DESIGN

This will be a randomized, placebo-controlled, double-blind, parallel trial evaluating the efficacy of dexamethasone prophylaxis in reducing the incidence of AMS and improving exercise performance in COPD patients travelling to altitude. The study design is shown in figure 1.

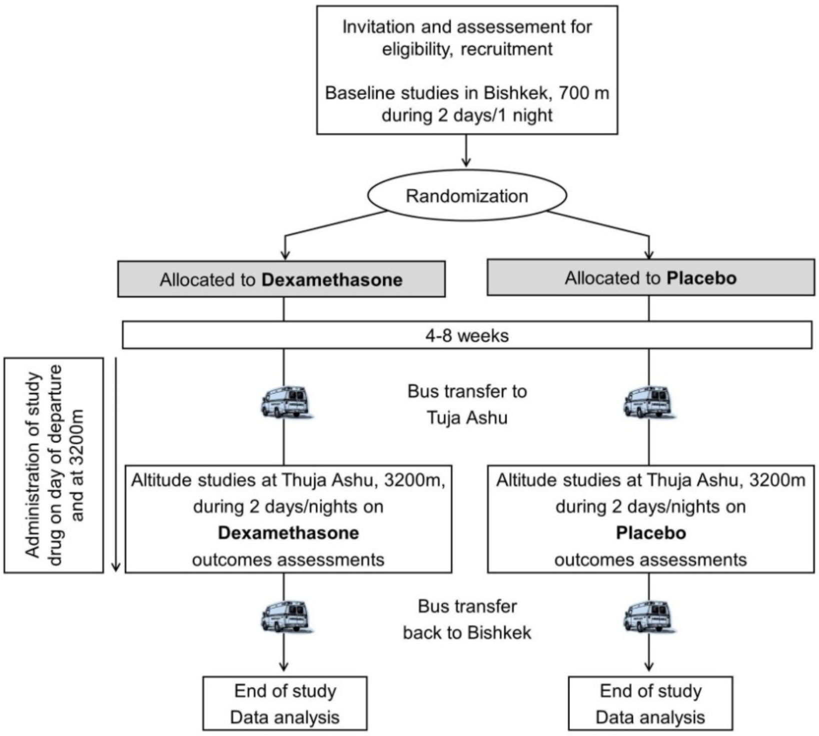

Figure 1. Study protocol. Baseline examination takes place in Bishkek (700 m). Within 4-8 weeks participants will be transferred by bus to the Tuja Ashu high altitude clinic (3200 m), 180 km north of Bishkek to stay there during 2 days and nights. Subsequently, participants will travel back to Bishkek. Study drugs (dexamathasone 2x4 mg/d or placebo) will be administered in the morning of the day of ascent and during the stay at altitude. Assessments will be performed at baseline and during the stay at altitude.

Daily assessments will include evaluation of AMS, 6MWD, and various other clinical and physiologic outcomes described above and shown in table 1 for studies in Bishkek, 490 m, and Tuja Ashu, 3200 m.

TABLE 1. SCHEDULE OF EXAMINATIONS, IN BISHKEK (700 M) AND TUJA AHSU (3200 M)

Day 1	Time	Activity
	13:00-16:00	arrival at study site
	16:00-18:30	history, questionnaires, clinical examination, spirometry
	18:30-20:00	dinner and rest

	20:00-22:00	montage of polygraphic equipment
	22:00-07:00	polygraphic sleep study
Day 2	07:00-08:00	clinical examination, arterial blood gas analysis
	08:00-09:00	breakfast
	09:00-13:00	questionnaires, vigilance test, balance test, spirometry, 6MWD
	13:00-18:00	Lunch, cerebral near-infrared spectroscopy and ultrasound blood flow monitoring, echocardiography, then rest
	18:00-20:00	Dinner
	20:00-22:00	montage of polygraphic equipment
	22:00-07:00	polygraphic sleep study
Day 3	07:00-08:00	clinical examination, arterial blood gas analysis
	08:00-09:00	breakfast
	09:00-12:30	questionnaires, vigilance test, balance test, spirometry, 6MWD
	12:30-13:30	Lunch, debriefing

Pre-study screening and safety considerations

During pre-study screening (recruitment examination in Bishkek), a detailed medical history will be obtained to identify any risk factors that may predispose to adverse altitude effects. Pulmonary function, ECG, maximal ergometry and pulse oximetry will be obtained. During travel and at study locations, a physician equipped with emergency material (including bottled oxygen, defibrillator, ambu-bag, drugs) will be present. A rescue plan for patients requiring evacuation to lower altitude will be prepared.

The high altitude clinic at Tuja Ashu is an institution with an infrastructure suitable for treatment of patients with medical problems including respiratory disease. In particular, rooms, electricity, water and heating are available at all times. An electricity generator will be available in case of unexpected loss of power. Two rooms will be dedicated to treatment of patients with any intercurrent illness. A physician and nurses who are not members of the research team will be responsible for medical care of study participants and investigators as needed. An ambulance car with driver will be stationed at the clinic in case evacuation to a lower altitude is required. The university hospital in Bishkek can be reached by a car drive of about 4 hours.

The main side effects of dexamethasone in the dose of 2x4 mg/d as used in the current study include sleep disturbances, high blood pressure, fluid retention, elevated blood glucose and peptic ulcer disease. The immunosuppressive effect of the drug is not relevant during administration for only 3 days. Glucocorticoids in doses equivalent to dexamethasone 8 mg/d are commonly administered to COPD patients to treat exacerbations. This treatment is generally well tolerated although patients should be monitored for the side effects mentioned above. This monitoring is ensured in the current study.

Based on values reported in healthy subjects[24,6,25] and on data obtained in patients with COPD, GOLD 2-3, in our own studies at Zurich and Davos[26] we have computed estimates of arterial blood gases and oxygen saturation in table 2. These calculations suggest that COPD patients, GOLD 1-2, will have moderate hypoxemia with an SaO_2 of 80-85% upon arrival at the study site in Tuja Ashu at 3200 m. Their blood gases are expected to fall between values observed in healthy

subjects at 2590 m and 4559 m, respectively. At 4559 m, 53% of healthy subjects have been reported to suffer from AMS.[27] Therefore, we expect that a similar percentage of COPD patients will have AMS at 3200 m. Regular clinical checks of health status and pulse oximetry, and daily arterial blood gas analysis will assure that any adverse health effects requiring intervention will be detected. Participants will be withdrawn from the study if the safety endpoints listed in table 3 are reached.

TABLE 2. ARTERIAL OXYGEN PARTIAL PRESSURE AND OXYGEN SATURATION

	Healthy subjects			COPD, GOLD 2-3 Zurich and Davos		COPD, GOLD 1-2 Tuja Ashu
Altitude, m	490	2590	4559	490	2590	3200
Barometric pressure, kPa	96.0	70.7	60.5	96.0	75.8	70.7
PiO2 (PB*.21-PH_2O), kPa	18.8	14.6	11.3	18.8	14.6	13.5
PaO_2, kPa	12.0	8.9	5.7	8.9	6.8	6.5
SaO_2, %	97	93	81	94	89	84
$PaCO_2$, kPa	4.8	3.9	4.0	5.5	4.9	4.3
$PAaO_2$, kPa	0.9	0.8	1.5	3.1	1.7	1.7

PiO_2 =PB*.21-PH2O; values in healthy subjects derived from [24,6,25]; values in COPD patients at 490 and 2590 m measured in own studies;[26] estimates in COPD patients at 3200 m based on own studies in COPD at 2590 m, assuming moderate hyperventilation compared to healthy subjects and absence of Bohr effect that would increase SaO2.

TABLE 3. SAFETY ENDPOINTS IN THE DEXAMETHASONE TRIAL

- exacerbation of COPD: worsening of respiratory symptoms that are beyond normal day to day variation and require a change in treatment exceeding intensified bronchodilator inhalations
- severe hypoxemia (SpO_2 <75% at rest during room air breathing for >30 min, SpO_2 <70% at rest during room air breathing for >15 min)
- intercurrent illness (such as infection, new diseases, accidents, etc.) that cannot be relieved by simple measures such as paracetamol 3x500 mg/day, inhalation of bronchodilators.
- severe AMS (AMSc score >2), not releaved by paracetamol 1 g bid
- dyspnea or discomfort at rest requiring treatment with oxygen
- chest pain or ECG signs of cardiac ischemia
- systolic blood pressure >200 mmHg, diastolic blood pressure >110 mmHg
- new onset neurologic impairment
- Any condition that requires study withdrawal according to the decision of the independent physician responsible for medical care of participants

DATA ANALYSIS AND STATISTICS

Primary outcomes

- Cumulative incidence of AMS (AMSc score >0.7) or of reaching any of the safety endpoints listed in table 3 during the stay at 3200 m

Secondary outcomes

- Incidence of AMS on day 1, 2 and 3 at 3200 m
- environmental symptoms cerebral score (AMSc), Lake Louise AMS score
- NYHA functional class
- Epworth sleepiness score, Karolinksa and Stanford Sleepiness scale, visual analog scale of sleep quality
- 6MWD
- Blood pressure, pulse rate
- Arterial blood gases
- Spirometry (FEV1, FVC in absolute units and in % predicted)
- Results of respiratory sleep studies: AHI, mean oxygen saturation, oxygen desaturations >3%
- Echocardiographic variables of right and left ventricular dimensions and function
- Postural control assessed by a balance platform
- Cerebral tissue oxygenation and middle cerebral artery blood flow velocity measured by near-infrared spectroscopy of the brain and Doppler ultrasound, respectively

Statistics and sample size estimation

Data will be summarized by means (SD) and medians (quartiles) for normal and non-normal distributions. Effects of HAPH and time, and of treatment and altitude will be evaluated by ANOVA and by multiple regression.

Sample size estimation. According to a study in the Alps, 53% of healthy subjects ascending to 4559 m are expected to suffer from AMS.[27] Based on estimates of the PaO2 in COPD patients at 3200 m (table 2) and a susceptibility to AMS of COPD patients at least as high as that in healthy subjects we estimate an incidence of AMS (AMSc score >0.7) of 60% in COPD patients at 3200 m. In order to reduce the AMS incidence (primary outcome) by half (to 30%), a sample size of 100 is required taking into account a drop-off rate of 15%.

4. Significance of the project

COPD is a highly prevalent disorder responsible for significant morbidity and mortality worldwide. Many COPD patients in Switzerland and elsewhere travel to altitude to pursue professional or recreational activities or because they are referred to an altitude clinic for rehabilitation. The proposed randomized, placebo-controlled trial evaluating dexamethasone prophylaxis as a means to prevent altitude related adverse health effects in COPD patients will provide valuable new scientific information. It will guide clinicians to decide whether such treatment should be prescribed to COPD patients travelling to altitude. Moreover, the study will also help to better appreciate the role of treating COPD patients living at low altitude during a stay in a high altitude rehabilitation clinic. As an institution that provides care for patients with COPD including oxygen therapy the Lunge Zürich might be particularly interested in the data obtained in the proposed project.

References

1. Latshang TD et al. Effect of travelling to 2590m on 6-minute walk distance, blood gases and symptoms of acute mountain sickness in COPD patients. Eur Respir J 2014.

2. Furian M et al. Exercise performance in patients with chronic obstructive lung disease at 2590 m. Interantional Hypoxia Conference 2015.

3. Latshang TD et al. Breathing and sleep distrubances in lowlanders with COPD travelling to 2590 m. Eur Respir J 2015.

4. Ulrich S et al. Right and left ventricular dysfunction in COPD patients travelling to altitude. Interantional Hypoxia Conference 2015.

5. Latshang TD et al. Effect of acetazolamide and autoCPAP therapy on breathing disturbances among patients with obstructive sleep apnea syndrome who travel to altitude: a randomized controlled trial. JAMA 2012;308:2390-2398.

6. Nussbaumer-Ochsner Y et al. Patients with obstructive sleep apnea syndrome benefit from acetazolamide during an altitude sojourn: a randomized, placebo-controlled, double-blind trial. Chest 2012;141:131-138.

7. Maggiorini M et al. Both tadalafil and dexamethasone may reduce the incidence of high-altitude pulmonary edema: a randomized trial. Ann Intern Med 2006;145:497-506.

8. Fischler M et al. Dexamethasone but not tadalafil improves exercise capacity in adults prone to high-altitude pulmonary edema. Am J Respir Crit Care Med 2009;180:346-352.

9. Niewoehner DE et al. Effect of systemic glucocorticoids on exacerbations of chronic obstructive pulmonary disease. Department of Veterans Affairs Cooperative Study Group. N Engl J Med 1999;340:1941-1947.

10. Jones PW et al. Development and first validation of the COPD Assessment Test. Eur Respir J 2009;34:648-654.

11. Borg E et al. A comparison between three rating scales for perceived exertion and two different work tests. Scand J Med Sci Sports 2006;16:57-69.

12. Mahler DA et al. Evaluation of clinical methods for rating dyspnea. Chest 1988;93:580-586.

13. Sampson JB et al. Procedures for the measurement of acute mountain sickness. Aviat Space Environ Med 1983;54:1063-1073.

14. Bloch KE et al. German version of the Epworth Sleepiness Scale. Respiration 1999;66:440-447.

15. Stadelmann K et al. Impaired postural control in healthy men at moderate altitude (1630 m and 2590 m). Data from a randomized trial. PLoS One 2015.

16. Bartsch P et al. Clinical practice: Acute high-altitude illnesses. N Engl J Med 2013;368:2294-2302.

17. Zheng CR et al. Inhaled budesonide and oral dexamethasone prevent acute mountain sickness. Am J Med 2014;127:1001-1009.

18. ATS. ATS statement: guidelines for the six-minute walk test. Am J Respir Crit Care Med 2002;166:111-117.

19. Miller MR et al. Standardisation of spirometry. Eur Respir J 2005;26:319-338.

20. Kojonazarov BK et al. Noninvasive and invasive evaluation of pulmonary arterial pressure in highlanders. Eur Respir J 2007;29:352-356.

21. Ulrich S et al. Cerebral Oxygenation in Patients With OSA: Effects of Hypoxia at Altitude and Impact of Acetazolamide. Chest 2014;146:299-308.

22. Maarsingh EJ et al. Respiratory muscle activity measured with a noninvasive EMG technique: technical aspects and reproducibility. J Appl Physiol 2000;88:1955-1961.

23. Bartsch P et al. Clinical practice: Acute high-altitude illnesses. N Engl J Med 2013;368:2294-2302.

24. Latshang TD et al. Are nocturnal breathing, sleep and cognitive performance impaired at moderate altitude (1630-2590m)? A randomized trial. Sleep 2013.

25. Grocott MP et al. Arterial blood gases and oxygen content in climbers on Mount Everest. N Engl J Med 2009;360:140-149.

26. Latshang TD et al. Effect of travelling to 2590m on 6-minute walk distance, blood gases and symptoms of acute mountain sickness in COPD patients. Respiration 2014.

27. Maggiorini M et al. Prevalence of acute mountain sickness in the Swiss Alps. BMJ 1990;301:853-855.

Effect of Dexamethasone on Sleep Related Breathing Disturbances in Patients with COPD at Altitude

NCT02450994

Data analysis plan

Contents

1. Introduction 3
2. Outcome measures 3
3. Sample size calculation 5
4. Analysis principles 5
5. Covariates 5
6. Data analyses 6
7. Missing data 7

1. Introduction

This is a randomized, double blinded, placebo-controlled parallel trial evaluating the effect of dexamethasone vs. placebo intake on sleep related breathing disturbances in patients with chronic obstructive pulmonary disease (COPD) travelling from 700 m to 3200 m and staying there for 2 nights (ClinicalTrials.gov: NCT02450994).

The main study hypotheses are:

1. **Main hypothesis:** in patients with COPD, GOLD grade 1-2, living at low altitude (700 m) dexamethasone taken before and during a stay at 3200 m improves mean nocturnal SpO2 compared to placebo.
2. Improvements of mean nocturnal oxygen saturation (according to 1) are related to a) an increase in respiratory center drive, b) improvement in pulmonary gas exchange as reflected in the PAaO2, c) stabilization of control of breathing as reflected in the ODI
3. Acute exposure to high altitude (3200 m) induces a reduction in mean nocturnal SpO2 related to a) an inadequate increase in respiratory center drive, b) impairment in pulmonary gas exchange as reflected in the PAaO2, c) destabilization of control of breathing as reflected in the ODI

Participants will undergo baseline examinations in Bishkek (700 m) and will travel to the rehabilitation clinic at Tuja-Ashu (3200 m) for 2 nights (2 days). Intake of dexamethasone (2 x 4 mg/d) or placebo will start on the day before ascent and will end with the last dose in the morning after the second night at 3200 m (7 tabl., 28 mg in total) according to randomization.

2. Outcome measures

2.1. Primary outcomes

The primary outcome will be the difference in altitude-induced changes in nocturnal mean arterial oxygen saturation (SpO_2) between dexamethasone and placebo group at 3200 m (night 1). SpO_2 will be monitored with a finger pulse oximeter during the whole night. For safety reasons, patients with severe nocturnal hypoxemia will receive supplemental oxygen (SpO_2<75% at rest during room air breathing for >30 min, SpO_2<70% at rest during room air breathing for >15 min) for the rest of the night.

2.2. Secondary outcomes

- Difference in altitude-induced changes in mean nocturnal SpO_2 between dexamethasone and placebo group (night 2 at 3200m)
- Difference in altitude-induced changes in oxygen desaturation index (ODI, Desaturation of $SpO_2 \geq 4\%$) between dexamethasone and placebo group (night 1 at 3200m)
- Difference in altitude-induced changes in ODI (Desaturation of $SpO_2 \geq 4\%$) between dexamethasone and placebo group (night 2 at 3200m)
- Difference in altitude-induced changes in apnea/hypopnea index (AHI) between dexamethasone and placebo group (night 1 at 3200m)
- Difference in altitude-induced changes in AHI between dexamethasone and placebo group (night 2 at 3200m)
- Difference in mean nocturnal SpO_2 between dexamethasone and placebo group (night 1 at 700m)
- Difference in ODI (Desaturation of $SpO_2 \geq 4\%$) between dexamethasone and placebo group (night 1 at 700m)
- Difference in AHI between dexamethasone and placebo group (night 1 at 700m)
- Difference in altitude-induced changes in psychomotor vigilance test reaction time between dexamethasone and placebo group (day 2 at 3200m)
- Difference in subjective sleepiness between dexamethasone and placebo group (day 2 at 700m)
- Difference in altitude-induced changes in subjective sleepiness between dexamethasone and placebo group (day 2 at 3200m)
- Difference in altitude-induced changes in cerebral tissue oxygenation (CTO) between dexamethasone and placebo group (night 1 at 3200m)
- Difference in altitude-induced changes in cerebral oxygen desaturation index (cODI, Desaturation of SpO2≥4% and CTO≥4%) between dexamethasone and placebo group (night 1 at 3200m)
- Difference in breathing frequency and periodic breathing assessed by spectral analysis between dexamethasone and placebo group (night 1 at 3200m)
- Difference in breathing frequency and periodic breathing assessed by spectral analysis between dexamethasone and placebo group (night 2 at 3200m)

- Difference between angle phase shift between thoracic and abdominal breathing excursions assessed by crosscorrelation between dexamethasone and placebo group (night 1 at 3200m)
- Difference between angle phase shift between thoracic and abdominal breathing excursions assessed by crosscorrelation between dexamethasone and placebo group (night 2 at 3200m)

3. Sample size calculation

The sample size estimation was based on the primary outcome of the trial evaluating effect of dexamethasone on acute mountain sickness (www.ClinicalTrials.gov NCT02450968). It revealed a minimal number of 100 participants. Based on these grounds and on data from previous trials, the current the trial was powered with 80% to detect a minimal difference in SpO_2 (primary outcome) of 1.7% (SD 3.0%), with a two-sided significance level of alpha 0.05.

4. Analysis principles

The primary analysis will be performed according to the intention to treat principle on the entire data set. Missing data will be replaced by multiple imputations. Multivariate regression analysis will be performed on outcomes adjusting for baseline value, age, gender and severity of airflow obstruction. Treatment effects will be estimated by mean differences and their 95% CI.
A per protocol analysis was performed on data from all participants with completed data. All statistical tests will be 2-sided at the 5% statistical significance level and any confidence intervals reported will be 95% confidence intervals.

5. Covariates

5.1. Minimization factors

The study is designed according to a randomized parallel trial for the intake of dexamethasone vs. placebo medication.
Randomization was performed by the STATA 13 plugin "rct_minim" according to Pocock SJ and Simon et al. Biometrics 1975;31:103-115. Minimization of randomization was

applied to equally balance the dexamethasone and placebo group in regard to the following:

1. Age, < or > 50 years
2. COPD severity, FEV_1 % predicted < or > 80 %
3. Gender, male or female

Investigators will be blinded during the analyses of all data. Exceptions are serious adverse events or other conditions that require immediate knowledge on dexamethasone or placebo to allow prevention of adverse health effects to the patient.

6. Data analyses

6.1. Baseline characteristics

Before analysis, data are anonymized by allocating a numerical code to each participant. Baseline factors will be assessed for distribution of data and summarized group using appropriate statistics (e.g. mean, standard deviation, median upper/lower quartiles, etc.). No formal statistical tests will be performed.

6.2. Primary outcome analysis

6.2.1 Patient flow

Number and causes of withdrawal are registered and will be presented in a study flow chart.

6.2.2 Subgroup analysis

Subgroup analysis will be performed in COPD GOLD 1 versus COPD GOLD 2.

Subgroup analysis will be performed between patients with and without nocturnal hypoxemia at 700 m (SpO_2 <90% for >30% of total sleep time).

6.2.3. Hypothesis testing (Efficacy analysis)

Completeness, plausibility and distribution of data will be assessed by inspection of numerical results and graphical display. Normality of distribution will be tested by the Shapiro Wilk test. Data will be summarized by means (SD) and medians (interquartile) for normal and non-normal distributions, respectively. The mean altitude-induced

changes in nocturnal SpO_2 at 3200 m will be computed and between group differences will be presented as mean differences (95% confidence intervals) or median differences (95% confidence intervals) according to the distribution of the data. Effects of acute altitude exposure and of dexamethasone will be further evaluated using multiple regression analysis controlling for baseline values, age, and gender. A p-value <0.05 will indicate statistical significance.

6.3. Secondary outcomes

6.3.1. Efficacy measures

Secondary outcomes will be tabulated and changes with altitude exposure and medication will be evaluated by comparison to baseline using regression analysis adjusting for baseline measurements, day at altitude, daytime, age and gender.

7. Missing data

In the intention to treat analysis, missing data will be replaced by multiple imputation using chained equations. In addition, a per protocol approach will be performed where missing values will not be replaced.
