# Supplementary material for: Effect of Dexamethasone on Nocturnal Oxygenation in Lowlanders With Chronic Obstructive Pulmonary Disease Traveling to 3100 Meters: A Randomized Clinical Trial
Source: JAMA Netw Open. 2019 Feb 22;2(2):e190067. doi: 10.1001/jamanetworkopen.2019.0067 (PMC6484579; doi:10.1001/jamanetworkopen.2019.0067)
Supplement: Supplement 2. — eMethods. Sleep Study Analysis and Additional Measurements eTable 1. Altitude-Induced Changes in Selected Variables eTable 2. Multivariable Regression Analysis of Predictors of Mean Nocturnal Oxygen Saturation (Spo2) at 3100 m eTable 3. Logistic Regression Analysis of Predictors (Including PaO2) of Premature Study Termination at 3100 m Due to Adverse Events eTable 4. Logistic Regression Analysis of Predictors (Including Spo2) of Early Study Termination at 3100 m Due to Adverse Events eFigure. Effect of Altitude and Dexamethasone on Apneas/Hypopneas and Blood Pressure eReferences [file jamanetwopen-2-e190067-s002.pdf]

Supplementary Online Content

Furian M, Lichtblau M, Aeschbacher SS, et al. Effect of dexamethasone on nocturnal oxygenation in lowlanders with chronic obstructive pulmonary disease traveling to 3100 meters: a randomized clinical trial. *JAMA Netw Open*. 2019;2(2):e190067. doi:10.1001/jamanetworkopen.2019.0067

eMethods. Sleep Study Analysis and Additional Measurements

eTable 1. Altitude-Induced Changes in Selected Variables

eTable 2. Multivariable Regression Analysis of Predictors of Mean Nocturnal Oxygen Saturation (SpO_2) at 3100 m

eTable 3. Logistic Regression Analysis of Predictors (Including PaO_2) of Premature Study Termination at 3100 m Due to Adverse Events

eTable 4. Logistic Regression Analysis of Predictors (Including SpO_2) of Early Study Termination at 3100 m Due to Adverse Events

eFigure. Effect of Altitude and Dexamethasone on Apneas/Hypopneas and Blood Pressure

eReferences

This supplementary material has been provided by the authors to give readers additional information about their work.

© 2019 Furian M et al. *JAMA Network Open.*

eMethods. Sleep Study Analysis and Additional Measurements

Sleep study analysis
Apneas/hypopneas were defined as a >50% reduction in nasal pressure swings or chest wall excursions during ≥10 seconds; obstructive events were scored if asynchronous or paradoxical chest wall excursions suggested continued effort or if a flattened inspiratory portion of the nasal pressure curve suggested flow limitation; central apneas/hypopneas were scored in the absence of criteria of obstructive events.[1,2] If central apneas/hypopneas occurred as part of a periodic breathing pattern for ≥3 consecutive cycles, events lasting ≥5 seconds were also scored. The apnea/hypopnea index, the oxygen desaturation index derived from pulse oximetry (>3% dips) and the cerebral tissue oxygen desaturation index (>3% dips in the cerebral near-infrared spectroscopy signal)[3] were computed as the mean number of events per hour from lights-off to lights-on.

Additional measurements
Spirometry (EasyOne, NDD, Zurich, Switzerland) was performed according to published standards. The Global Lung Function Initiative reference values for Caucasians were applied since specific references values for Kyrgyz have not been published.[4] Arterial blood gas analysis (RapidPoint 405, Siemens Healthcare Diagnostics GmbH, Zurich, Switzerland) were obtained from a radial artery blood sample. During the psychomotor vigilance test (PVT) subjects were sitting in a quiet room and had to press a button in response to light signals appearing at irregular intervals during 10 min while the reaction time was recorded.[5]

© 2019 Furian M et al. *JAMA Network Open.*

eTable 1. Altitude-Induced Changes in Selected Variables

	Night 1 at 3100 m vs. 760 m				Night 2 at 3100 m vs. 760 m			
**Variables**	**Placebo**	**P value**	**Dexamethasone**	**P value**	**Placebo**	**P value**	**Dexamethasone**	**P value**
Mean nocturnal SpO_2, %	-8 (-9 to -7)	<.001	-5 (-6 to -5)	<.001	-7 (-7 to -6)	<.001	-4 (-5 to -4)	<.001
ODI, events/h	23.7 (19.2 to 28.2)	<.001	8.3 (4.2 to 12.5)	<.001	21.0 (16.4 to 25.5)	<.001	5.3 (1.1 to 9.5)	.013
AHI, events/h	19.1 (14.3 to 24.0)	<.001	0.5 (-4.0 to 5.0)	.838	16.3 (11.4 to 21.2)	<.001	-1.4 (-5.9 to 3.2)	.554
Central AHI, events/h	18.4 (13.6 to 23.2)	<.001	6.4 (2.0 to 10.9)	.004	18.5 (13.7 to 23.3)	<.001	6.4 (1.9 to 10.9)	.005
Obstructive AHI, events/h	0.7 (-2.9 to 4.3)	.700	-6.0 (-9.3 to -2.7)	<.001	-2.3 (-5.9 to 1.3)	.219	-7.8 (-11.1 to -4.4)	<.001
Subjective sleep quality, %[a]	-7 (-15 to 2)	.118	4 (-4 to 12)	.303	-3 (-11 to 5)	.494	9 (1 to 16)	.025
Morning MAP, mm Hg	1 (-2 to 4)	.607	-4 (-7 to -2)	.001	3 (1 to 6)	.019	0 (-3 to 3)	.966

Abbreviations: Mean differences (95% confidence interval); AHI, apnea/hypopnea index; MAP, mean arterial pressure; ODI, oxygen desaturation index, >3% dips in SpO_2; SpO_2, arterial oxygen saturation measured by pulse oximetry.

[a]Assessed subjectively. Subjective sleep quality was assessed by a 100-mm visual analog scale ranging from 0 (extremely bad) to 100 (excellent).

© 2019 Furian M et al. *JAMA Network Open.*

eTable 2. Multivariable Regression Analysis Of Predictors Of Mean Nocturnal Oxygen Saturation (SpO_2) at 3100 m

**Predictors**	**Coefficient**	**SE**	**P value**	**95% confidence interval**
Reference: Night at 760 m in the placebo group				
Night 1 at 3100 m vs. 760 m with placebo	-8.42	0.28	<.001	-8.96 to -7.87
Night 2 at 3100 m vs. 760 m with placebo	-6.75	0.29	<.001	-7.31 to -6.18
Dexamethasone vs. Placebo at 760 m	-0.21	0.39	.584	-0.97 to 0.55
Interaction Night*dexamethasone				
Night 1 at 3100 m with dexamethasone	2.68	0.39	<.001	1.91 to 3.44
Night 2 at 3100 m with dexamethasone	2.46	0.40	<.001	1.68 to 3.24
Age, years	-0.03	0.19	.095	-0.07 to 0.01
Female vs. Male	0.60	0.56	.289	-0.51 to 1.70
760 m: PaO_2, kPa	0.50	0.17	.004	0.17 to 0.84
760 m: $PaCO_2$, kPa	-1.15	0.38	.002	-1.89 to -0.41
760 m: FEV_1, %predicted	0.02	0.01	.056	0.00 to 0.04
760 m: Apnea/hypopnea index, 1/h	-0.03	0.01	.002	-0.05 to -0.01
760 m: Body mass index, kg/m^2	-0.06	0.04	.122	-0.14 to 0.02
Intercept	94.75	3.63	<.001	87.62 to 101.87

Abbreviations: $DAaPO_2$, alveolar-arterial PO_2 difference calculated according to Crapo et al.[6]; FEV_1, forced expiratory volume in the first second of expiration; $PaCO_2$, partial pressure of arterial CO_2; SE, standard error.

© 2019 Furian M et al. *JAMA Network Open.*

eTable 3. Logistic Regression Analysis of Predictors (Including PaO_2) of Premature Study Termination at 3100 m Due to Adverse Events

Predictors	Odds ratio	SE	P value	95% confidence interval
Dexamethasone vs. Placebo	0.05	0.06	.023	<0.01 to 0.65
Age, years	0.97	0.06	.591	0.85 to 1.09
Female vs. Male	omitted*			
760 m: PaO_2, kPa	0.26	0.15	.016	0.09 to 0.78
760 m: $PaCO_2$, kPa	1.73	2.21	.670	0.14 to 21.26
760 m: FEV_1, %predicted	0.98	0.03	.580	0.93 to 1.04
760 m: Body mass index, kg/m^2	0.67	0.12	.026	0.48 to 0.95

*sex was omitted from the regression since all adverse events were in males and none in females.
According to this analysis, the risk of experiencing an adverse event that required an intervention such as administration of oxygen or medication and premature study termination was increased by a mean of 3.84 (95% CI, 1.28 to 11.11) for each kPa reduction in PaO_2 at 760 m and this risk was reduced 20 times (95% CI, 4.34 to >100) by preventive dexamethasone treatment.
Abbreviations: FEV_1, forced expiratory volume in the first second of expiration; $PaCO_2$, partial pressure of arterial CO_2; PaO_2, partial pressure of arterial O_2; SE,standard error.

© 2019 Furian M et al. *JAMA Network Open.*

eTable 4. Logistic Regression Analysis of Predictors (Including SpO_2) of Early Study Termination at 3100 M Due to Adverse Events

Predictors	Odds ratio	SE	P value	95% confidence interval
Dexamethasone vs. Placebo	0.08	0.10	.032	0.01 to 0.81
Age, years	0.98	0.05	.751	0.88 to 1.10
Female vs. Male	omitted*			
760 m: daytime SpO_2, %	0.92	0.23	.749	0.56 to 1.51
760 m: $PaCO_2$, kPa	0.99	1.18	.991	0.10 to 10.21
760 m: FEV_1, %predicted	0.97	0.02	.206	0.92 to 1.02
760 m: Body mass index, kg/m^2	0.73	0.10	.028	0.55 to 0.97

*Sex was omitted from the regression since all adverse events were in males and none in females.
Abbreviations: FEV_1, forced expiratory volume in the first second of expiration; $PaCO_2$, partial pressure of arterial CO_2; SE, standard error; SpO_2, arterial oxygen saturation assessed by pulse oximetry.

© 2019 Furian M et al. *JAMA Network Open.*

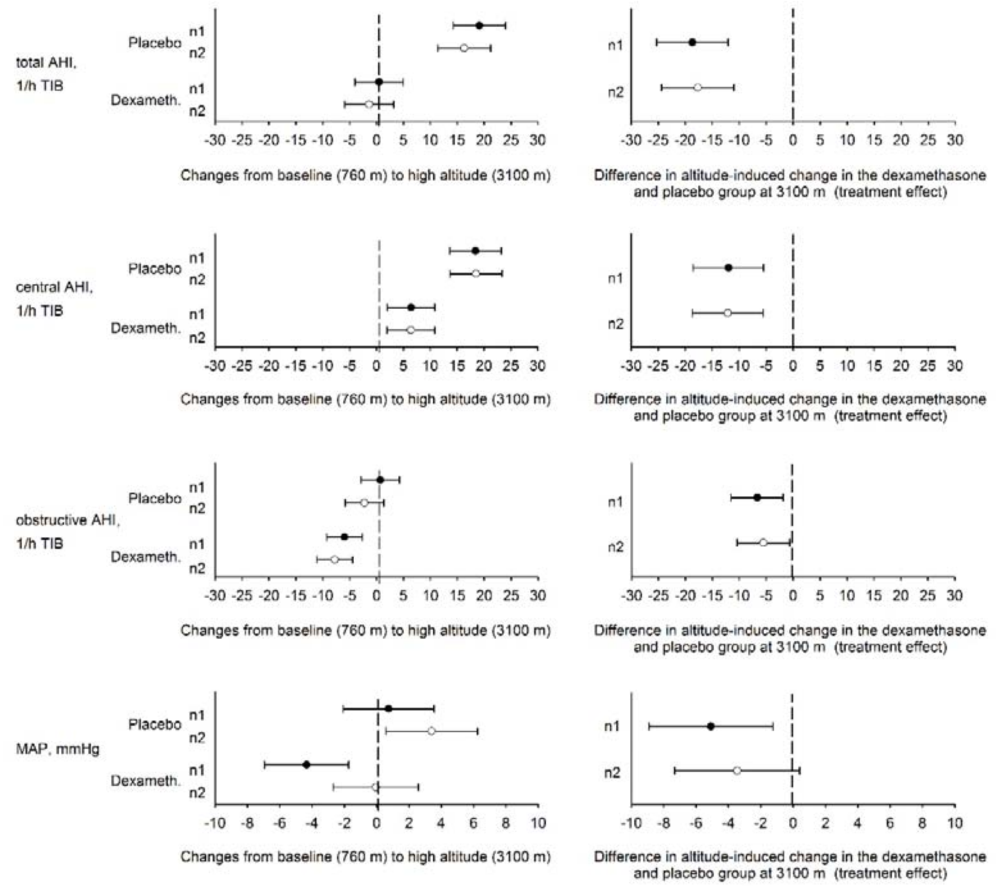

eFigure: Effect of Altitude and Dexamethasone on Apneas/Hypopneas and Blood Pressure. The left panels show mean differences and 95% confidence intervals of altitude-induced changes in the first night (closed circles) and second night (open circles) at 3100 m compared to the corresponding baseline examination at 760 m in patients receiving dexamethasone and placebo. The right panel show the mean differences and 95% confidence intervals in altitude-induced changes measured at 3100 m between patients receiving dexamethasone and placebo (treatment effect of dexamethasone). n1, n2, first and second night at 3100 m; AHI, apnea/hypopnea index; MAP, mean arterial blood pressure.

© 2019 Furian M et al. *JAMA Network Open.*

eReferences

1. Nussbaumer-Ochsner Y, Schuepfer N, Ulrich S, Bloch KE. Exacerbation of sleep apnoea by frequent central events in patients with the obstructive sleep apnoea syndrome at altitude: a randomised trial. *Thorax.* 2010;65(5):429-435.
2. Latshang TD, Nussbaumer-Ochsner Y, Henn RM, et al. Effect of acetazolamide and autoCPAP therapy on breathing disturbances among patients with obstructive sleep apnea syndrome who travel to altitude: a randomized controlled trial. *JAMA.* 2012;308(22):2390-2398.
3. Ulrich S, Nussbaumer-Ochsner Y, Vasic I, et al. Cerebral oxygenation in patients with OSA: effects of hypoxia at altitude and impact of acetazolamide. *CHEST.* 2014;146(2):299-308.
4. Quanjer PH, Stanojevic S, Cole TJ, et al. Multi-ethnic reference values for spirometry for the 3-95-yr age range: the global lung function 2012 equations. *Eur Respir J.* 2012;40(6):1324-1343.
5. Basner M, Dinges DF. Maximizing sensitivity of the psychomotor vigilance test (PVT) to sleep loss. *SLEEP.* 2011;34(5):581-591.
6. Crapo RO, Jensen RL, Hegewald M, Tashkin DP. Arterial blood gas reference values for sea level and an altitude of 1,400 meters. *Am J Respir Crit Care Med.* 1999;160(5):1525-1531.

© 2019 Furian M et al. *JAMA Network Open.*
