# Supplementary material for: Effect of Dexamethasone on Nocturnal Oxygenation in Lowlanders With Chronic Obstructive Pulmonary Disease Traveling to 3100 Meters: A Randomized Clinical Trial
Source: JAMA Netw Open. 2019 Feb 22;2(2):e190067. doi: 10.1001/jamanetworkopen.2019.0067 (PMC6484579; doi:10.1001/jamanetworkopen.2019.0067)
Supplement: Supplement 3. — Data Sharing Statement [file jamanetwopen-2-e190067-s003.pdf]

Data Sharing Statement

Furian. Effect of Dexamethasone on Nocturnal Oxygenation in Lowlanders With Chronic Obstructive Pulmonary Disease Traveling to 3100 Meters. *JAMA Netw Open*. Published February 22, 2019. 10.1001/jamanetworkopen.2019.0067

Data
Data available: No

Additional Information
Explanation for why data not available: Data can be accessed by request.
